# Supplementary material for: Dual Targeting Ligands—Histamine H3 Receptor Ligands with Monoamine Oxidase B Inhibitory Activity—In Vitro and In Vivo Evaluation
Source: Pharmaceutics. 2022 Oct 13;14(10):2187. doi: 10.3390/pharmaceutics14102187 (PMC9607599; doi:10.3390/pharmaceutics14102187)
Supplement: Supplementary file 1 [file pharmaceutics-14-02187-s001.zip › Supplementary S1_Table S1.pdf]

Table S1 – Structures of compounds **4-32**.

**Comp.**	Structure
**4**	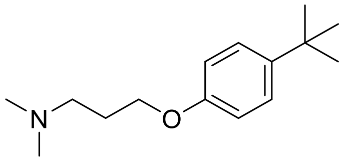
**5**	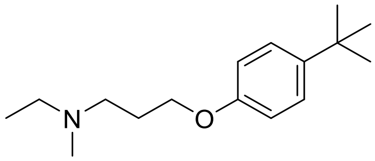
**6**	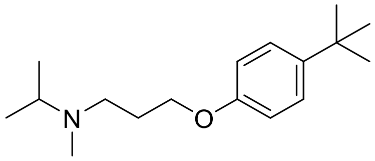
**7**	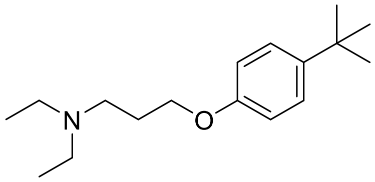
**8**	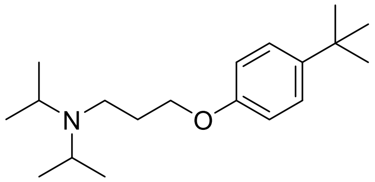
**9**	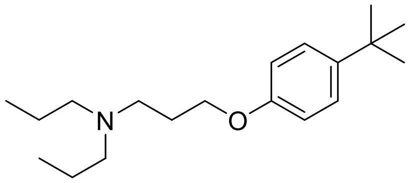
**10**	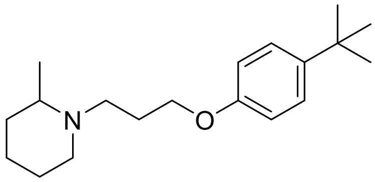
**11**	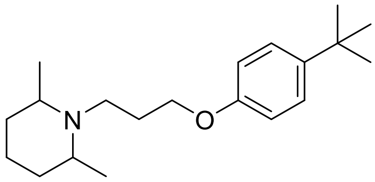
**12**	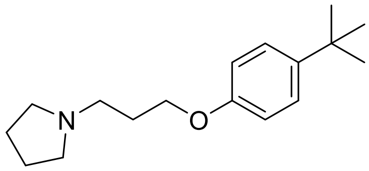

**Comp.**	Structure
**19**	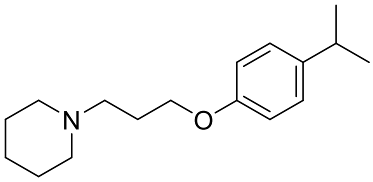
**20**	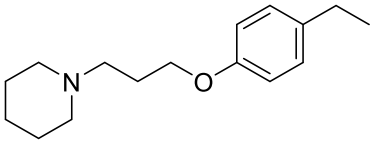
**21**	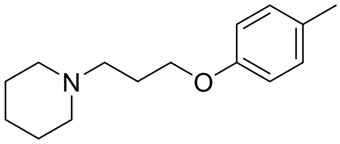
**22**	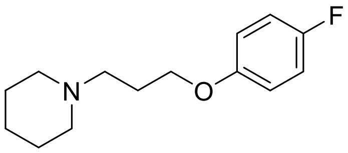
**23**	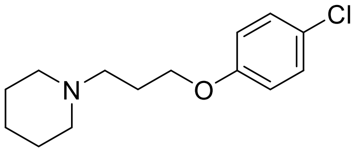
**24**	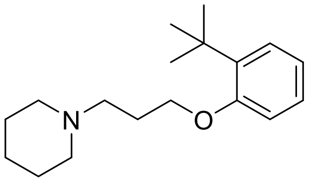
**25**	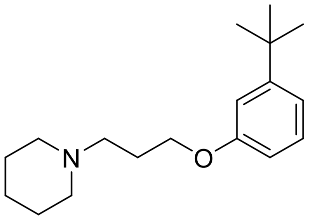
**26**	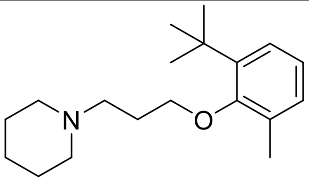
**27**	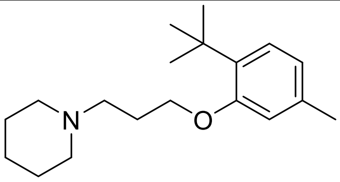

**13**	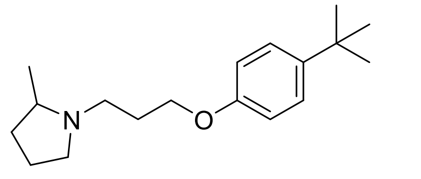
**14**	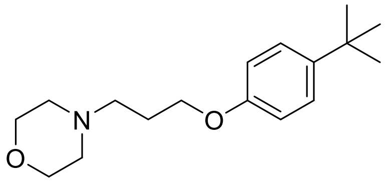
**15**	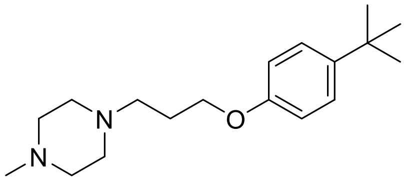
**16**	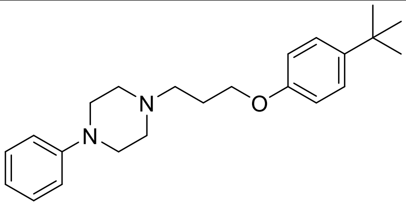
**17**	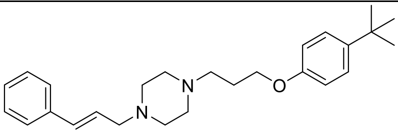
**18**	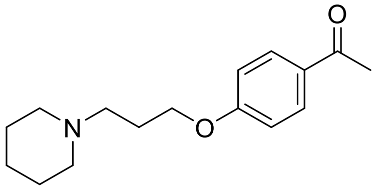

**28**	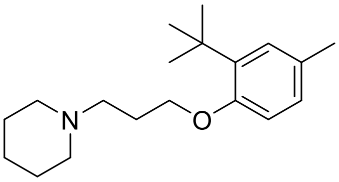
**29**	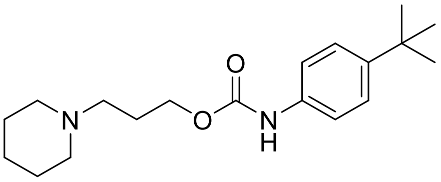
**30**	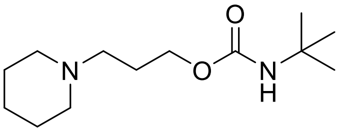
**31**	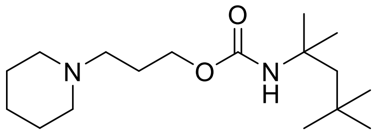
**31**	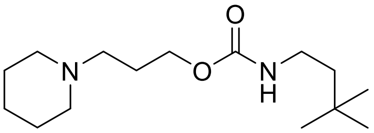
