# Supplementary material for: Dual Targeting Ligands—Histamine H3 Receptor Ligands with Monoamine Oxidase B Inhibitory Activity—In Vitro and In Vivo Evaluation
Source: Pharmaceutics. 2022 Oct 13;14(10):2187. doi: 10.3390/pharmaceutics14102187 (PMC9607599; doi:10.3390/pharmaceutics14102187)
Supplement: Supplementary file 1 [file pharmaceutics-14-02187-s001.zip › Supplementary S2.pdf]

Dual targeting ligands - histamine H_3 receptor ligands with monoamine oxidase B inhibitory activity – *in vitro* and *in vivo* evaluation

Dorota Łażewska[1*], Agata Siwek[2], Agnieszka Olejarz-Maciej[1], Agata Doroz-Płonka[1], Anna Wiktorowska-Owczarek[3], Marta Jóźwiak-Bębenista[3], David Reiner-Link[4], Annika Frank[4], Wioletta Sromek-Trzaskowska[1], Ewelina Honkisz-Orzechowska[1], Ewelina Królicka[1], Holger Stark[4], Marek Wieczorek[5], Waldemar Wagner[6,7], Katarzyna Kieć-Kononowicz[1], Anna Stasiak[6*]

[1]Department of Technology and Biotechnology of Drugs, Faculty of Pharmacy, Jagiellonian University Medical College in Kraków, Medyczna 9 Str., 30-688 Kraków, Poland; a.doroz-plonka@uj.edu.pl (A.D.-P.); agnieszka.olejarz@uj.edu.pl (A.O.-M.); wiola.diana@wp.pl (W.S.-T.); ewelina.honkisz@uj.edu.pl (E.H.-O.); ewelina.krolicka@uj.edu.pl (E.K.); mfkonono@cyf-kr.edu.pl (K.K.-K.)

[2]Department of Pharmacobiology, Faculty of Pharmacy, Jagiellonian University Medical College in Kraków, Medyczna 9 Str, 30-688 Kraków, Poland; agat.siwek@uj.edu.pl (A.S.)

[3]Department of Pharmacology and Toxicology, Medical University of Lodz, Żeligowskiego 7/9 Str., 90-752 Łódź, Poland; anna.wiktorowska-owczarek@umed.lodz.pl (A.W.-O.); marta.jozwiak-bebenista@umed.lodz.pl (M.J.-B.)

[4]Institute of Pharmaceutical and Medicinal Chemistry, Heinrich Heine University Düsseldorf, Universitaetsstr. 1, Düsseldorf 40225, Germany; reiner@hhu.de (D.R.-L.); annika.frank@hhu.de (A.F.); stark@hhu.de (H.S.)

[5]Department of Neurobiology, Faculty of Biology and Environmental Protection, University of Lodz, Pomorska 141/143 Str., 90-236 Łódź, Poland; marek.wieczorek@biol.uni.lodz.pl (M.W.)

[6]Department of Hormone Biochemistry, Medical University of Lodz, Żeligowskiego 7/9 Str., 90-752 Łódź, Poland; waldemar.wagner@umed.lodz.pl (W.W.)

[7] Laboratory of Cellular Immunology, Institute of Medical Biology of Polish Academy of Sciences, 106 Lodowa Str., 93-232 Łódź, Poland (W.W.)

*****Correspondence: dorota.lazewska@uj.edu.pl (D.Ł.); anna.stasiak@umed.lodz.pl (A.St.)

^{1}H and ^{13}C NMR spectra of synthesized compounds

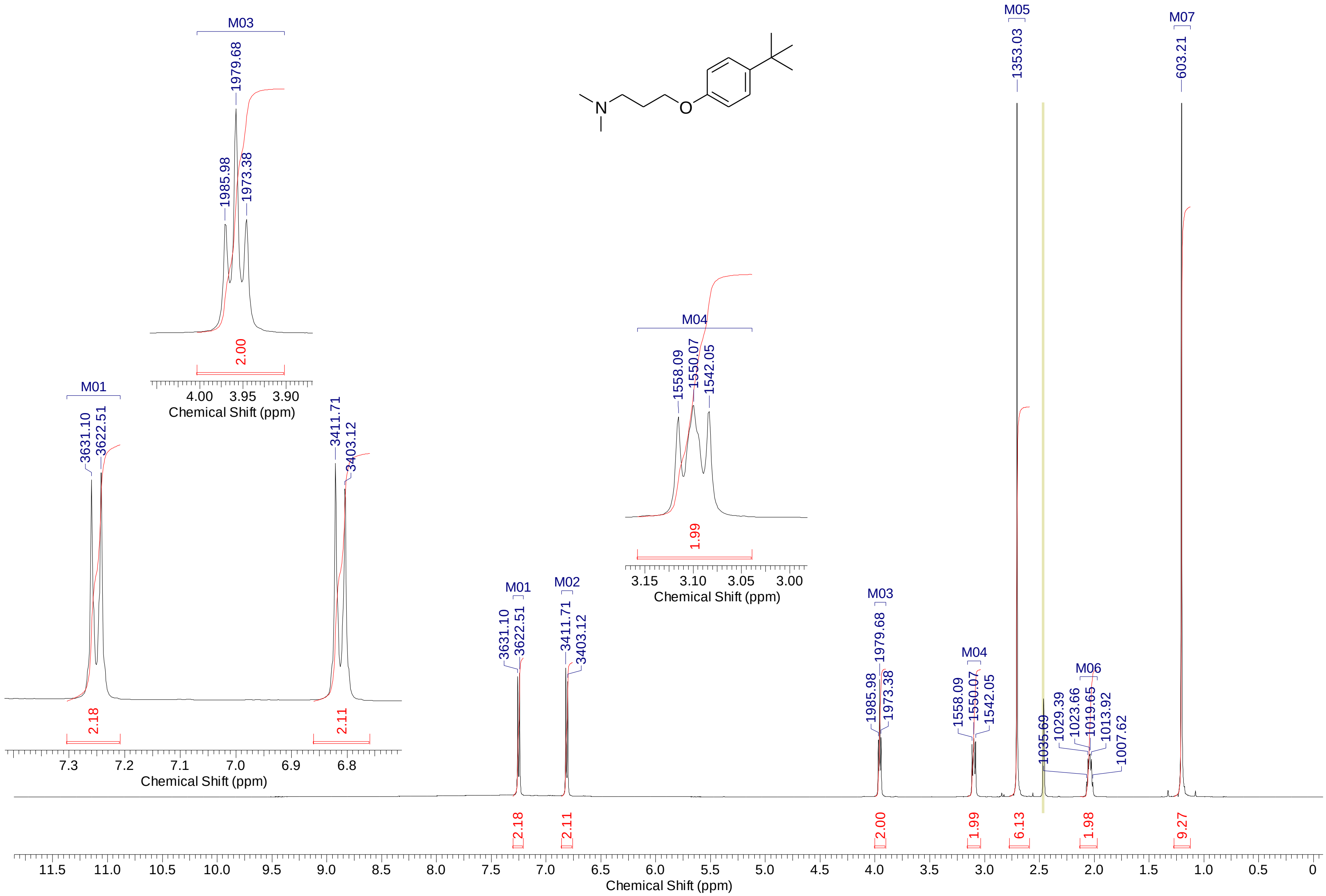

^{1}H NMR spectrum of 3-(4-(*tert*-butyl)phenoxy)-*N,N*-dimethylpropan-1-amine hydrogen oxalate (**4**)

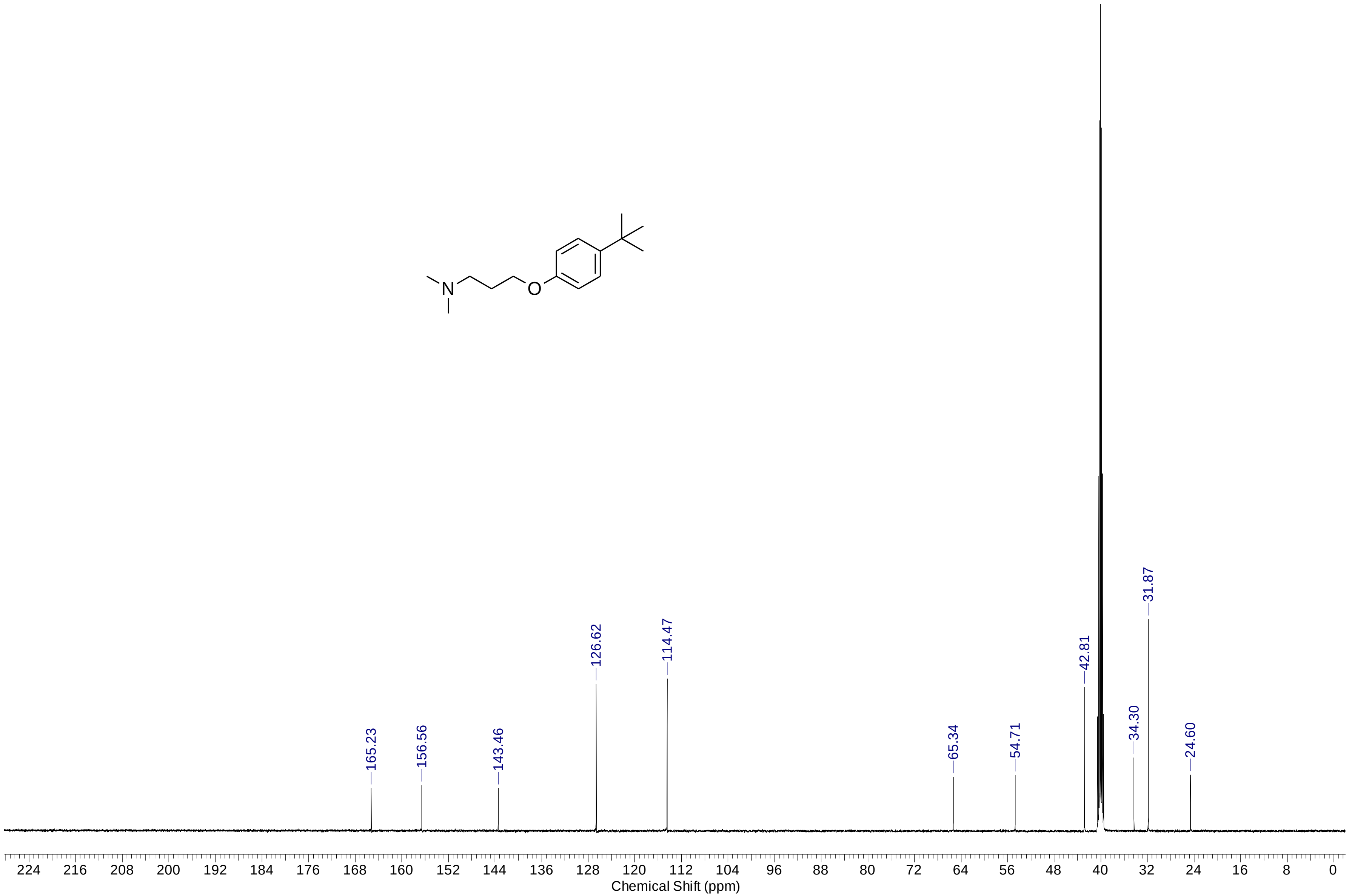

^{13}C NMR spectrum of 3-(4-(*tert*-butyl)phenoxy)-*N*,*N*-dimethylpropan-1-amine hydrogen oxalate (**4**)

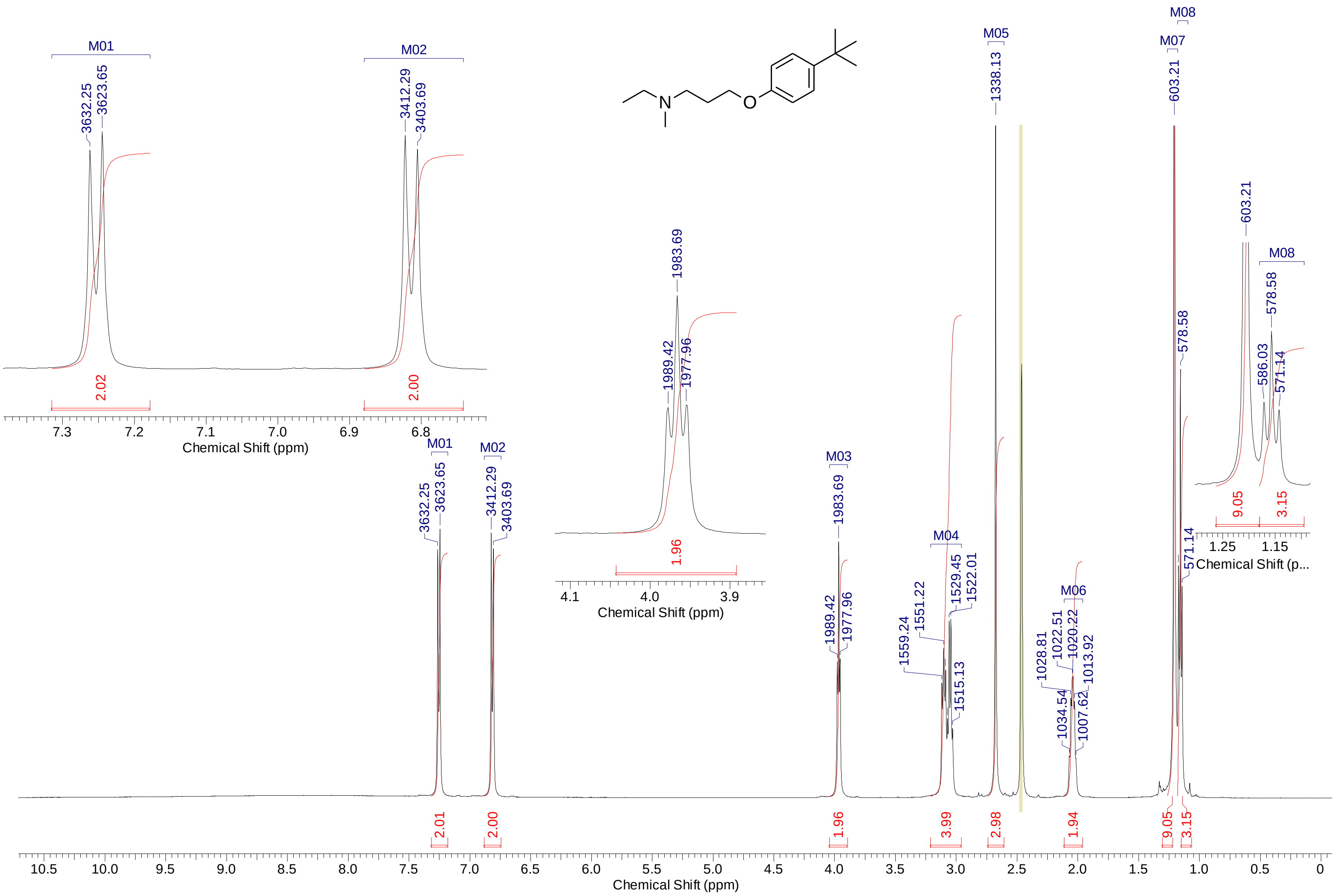

^{1}H NMR spectrum 3-(4-(*tert*-butyl)phenoxy)-*N*-isopropyl-*N*-methylpropan-1-amine hydrogen oxalate (**5**)

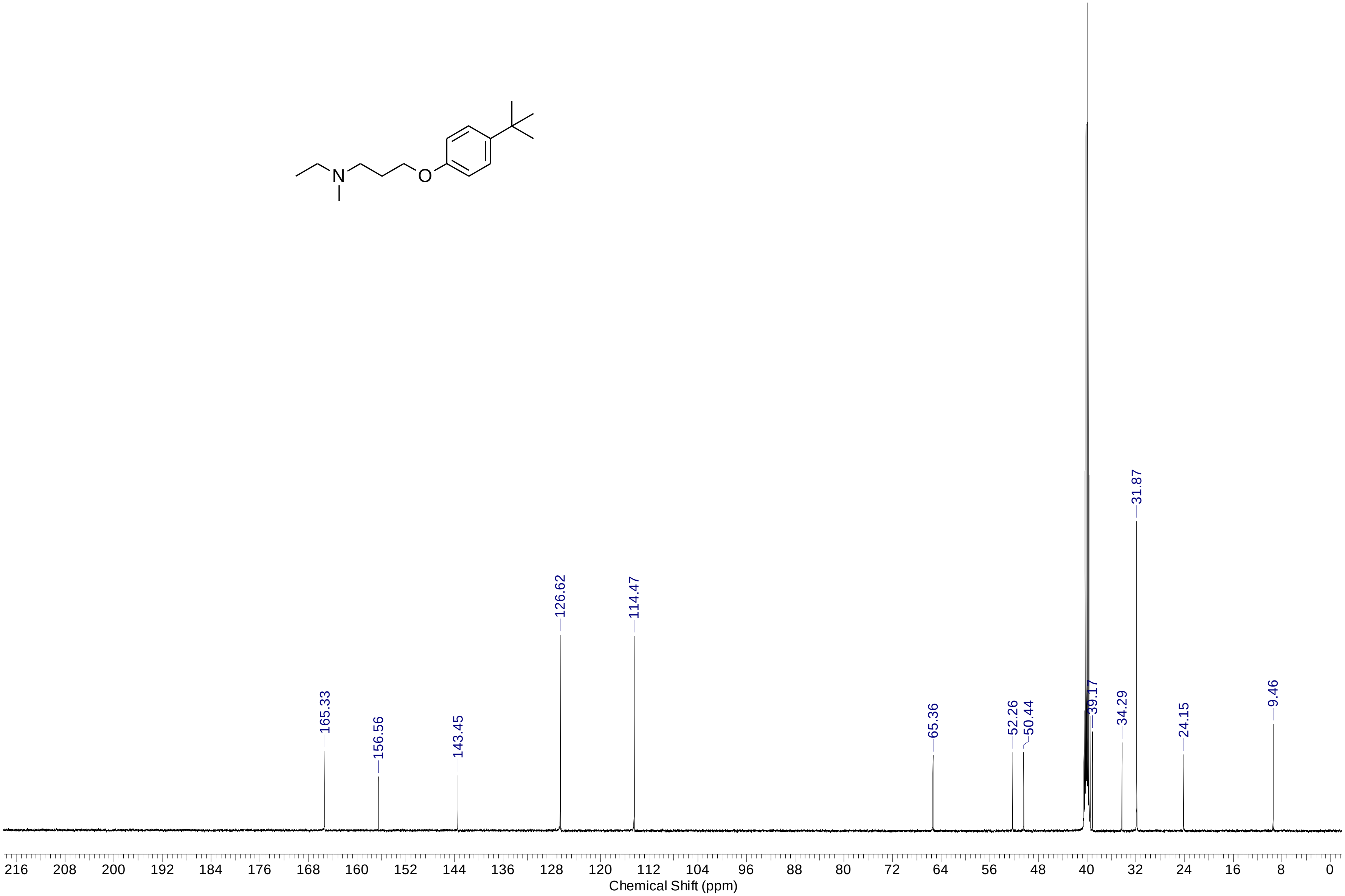

^{13}C NMR spectrum of 3-(4-(*tert*-butyl)phenoxy)-*N*-ethyl-*N*-methylpropan-1-amine hydrogen oxalate (**5**)

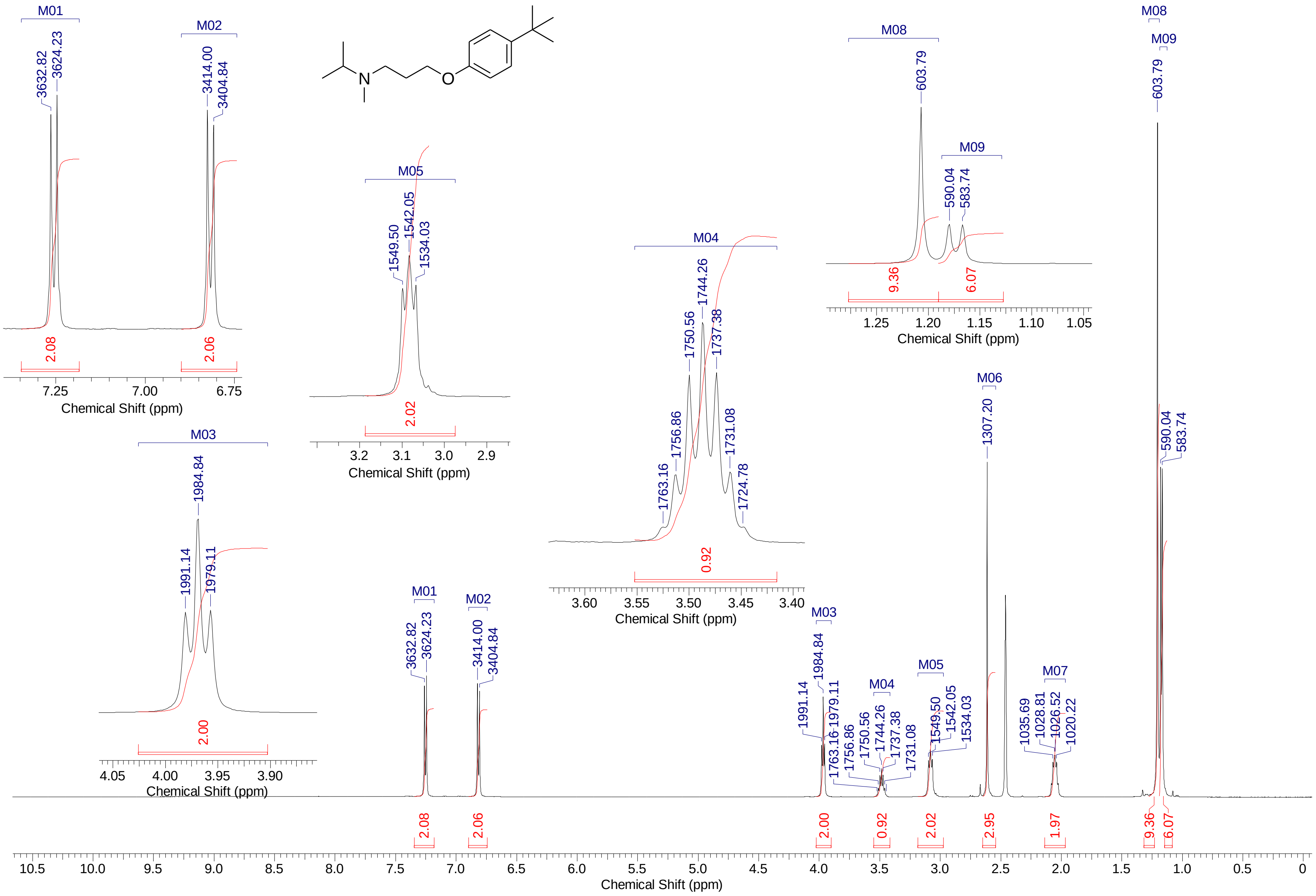

^{1}H NMR spectrum of 3-(4-(*tert*-butyl)phenoxy)-*N*-ethyl-*N*-methylpropan-1-amine hydrogen oxalate (**6**)

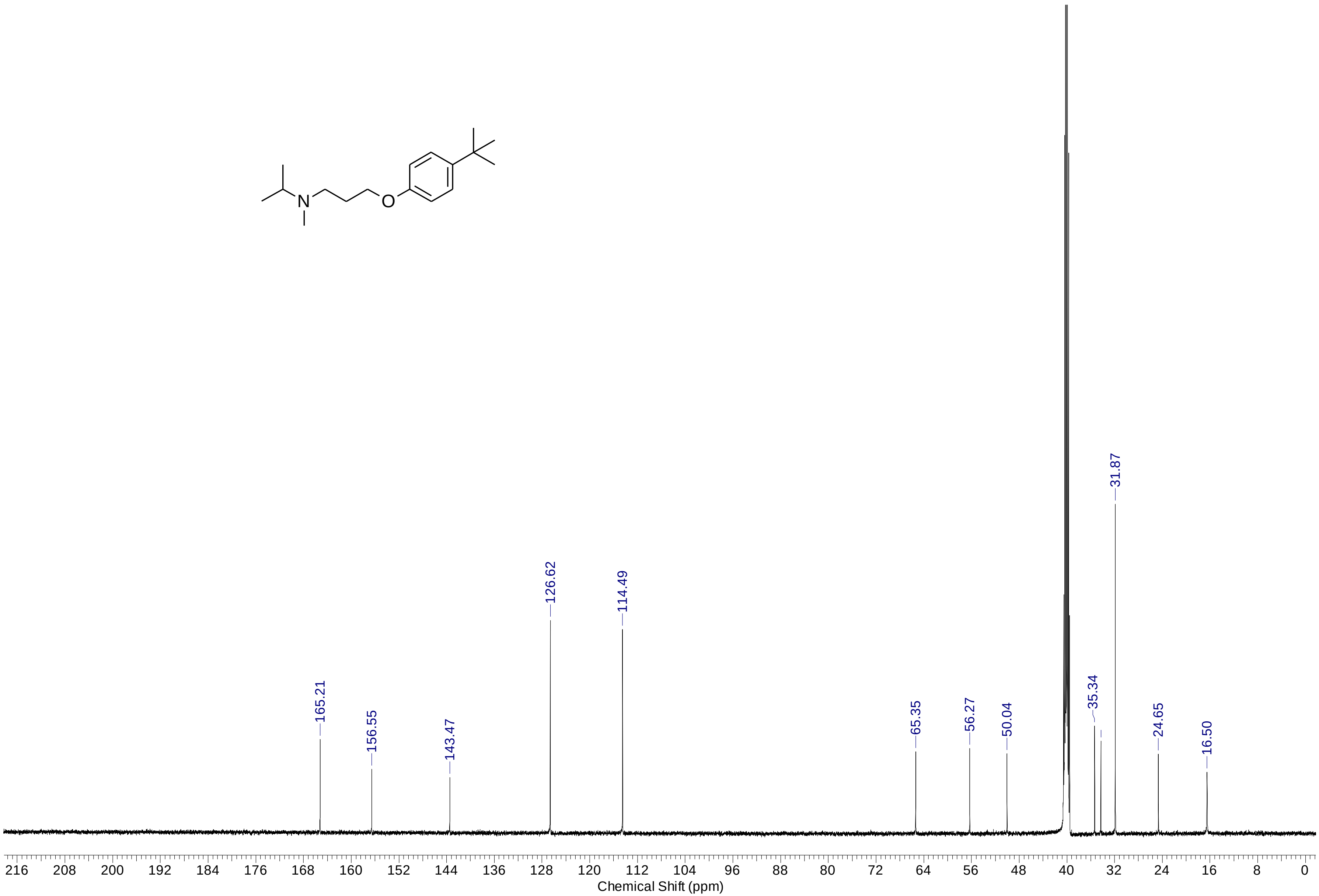

^{13}C NMR spectrum 3-(4-(*tert*-butyl)phenoxy)-*N*-isopropyl-*N*-methylpropan-1-amine hydrogen oxalate (**6**)

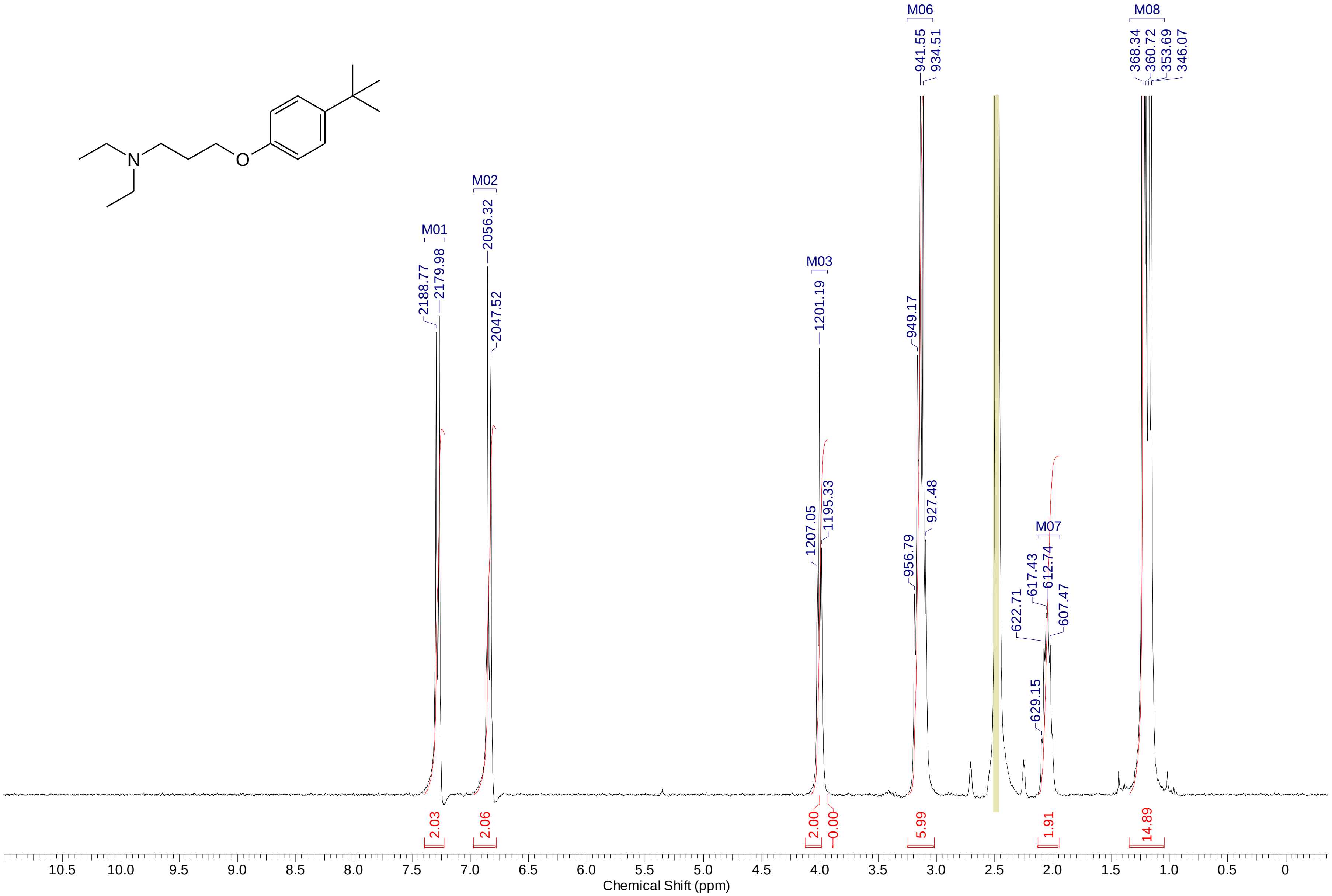

^{1}H NMR spectrum of 3-(4-(*tert*-butyl)phenoxy)-*N*,*N*-diethylpropan-1-amine hydrogen oxalate (**7**)

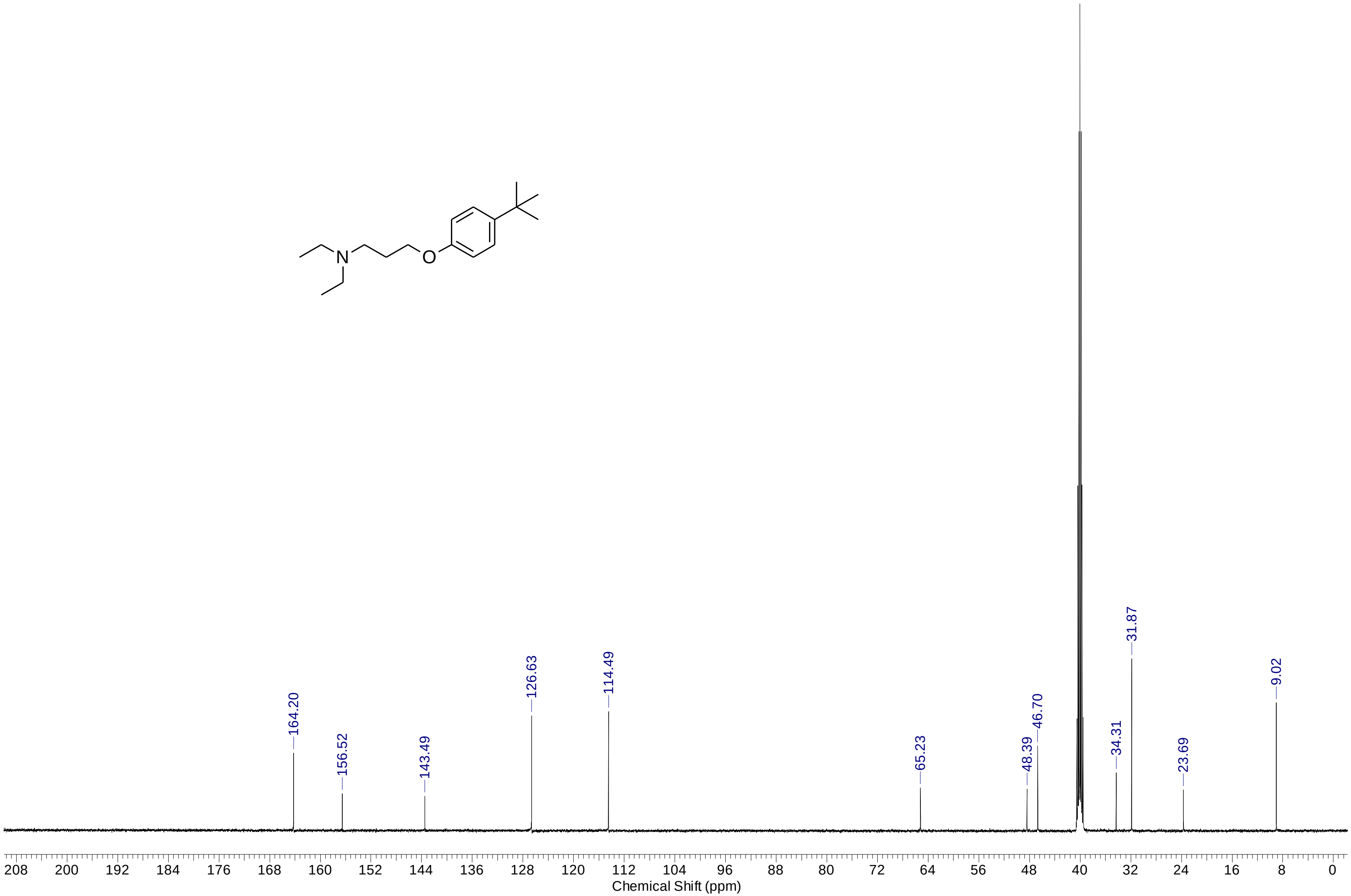

^{13}C NMR spectrum of 3-(4-(*tert*-butyl)phenoxy)-*N*,*N*-diethylpropan-1-amine hydrogen oxalate (**7**)

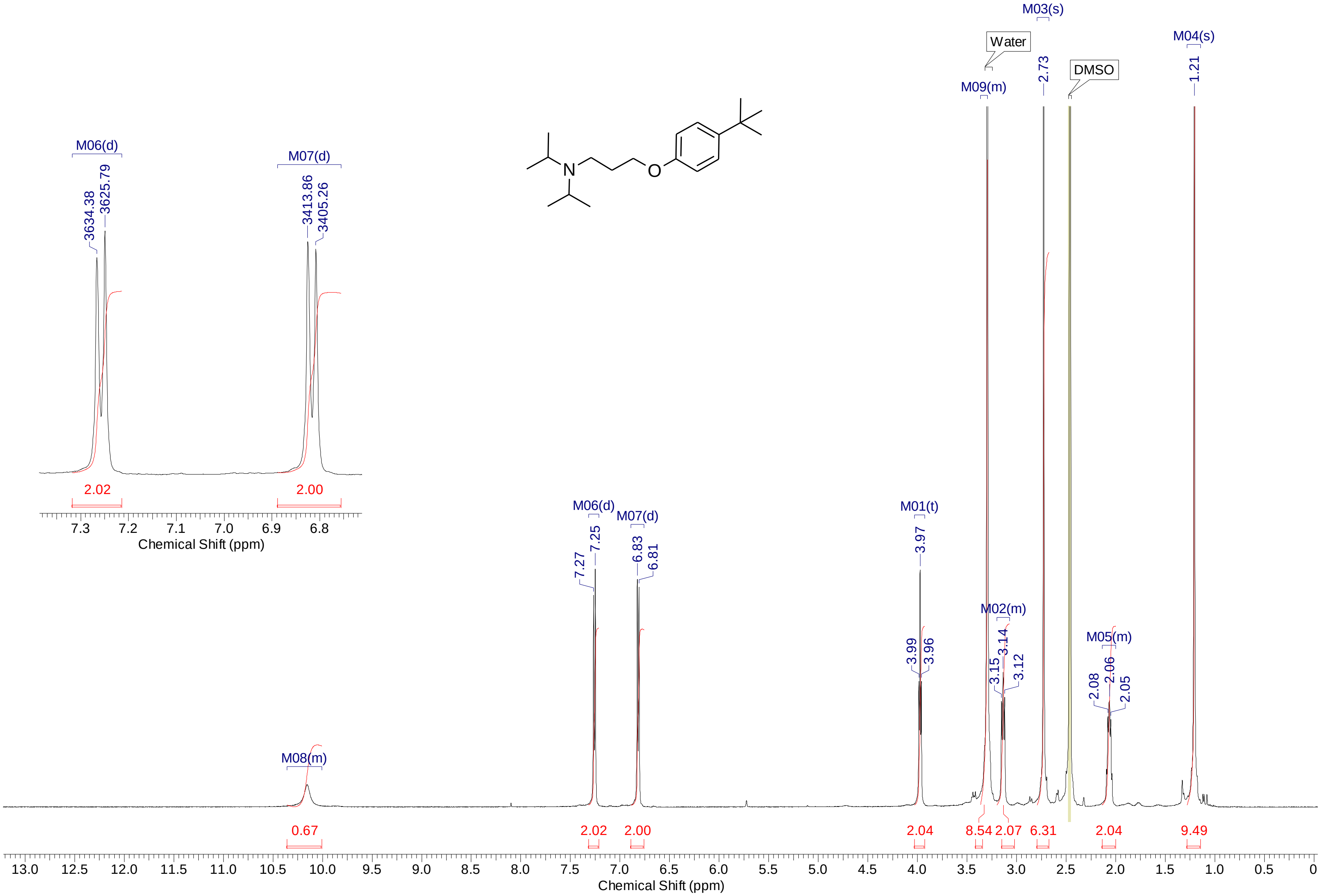

^{1}H NMR spectrum of 3-(4-(*tert*-butyl)phenoxy)-*N*,*N*-diisopropylpropan-1-amine hydrogen chloride (**8**)

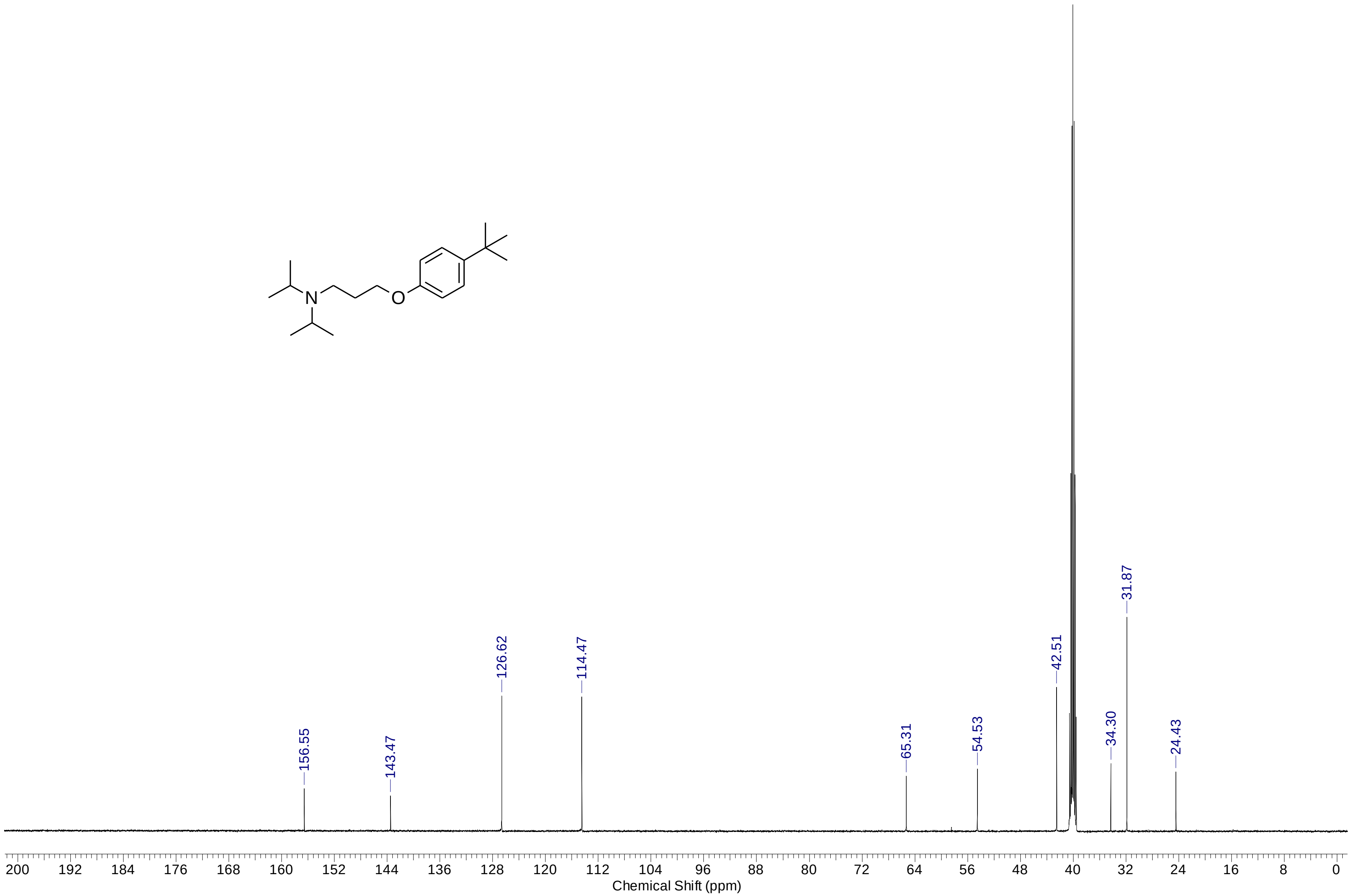

^{13}C NMR spectrum of 3-(4-(*tert*-butyl)phenoxy)-*N*,*N*-diisopropylpropan-1-amine hydrogen chloride (**8**)

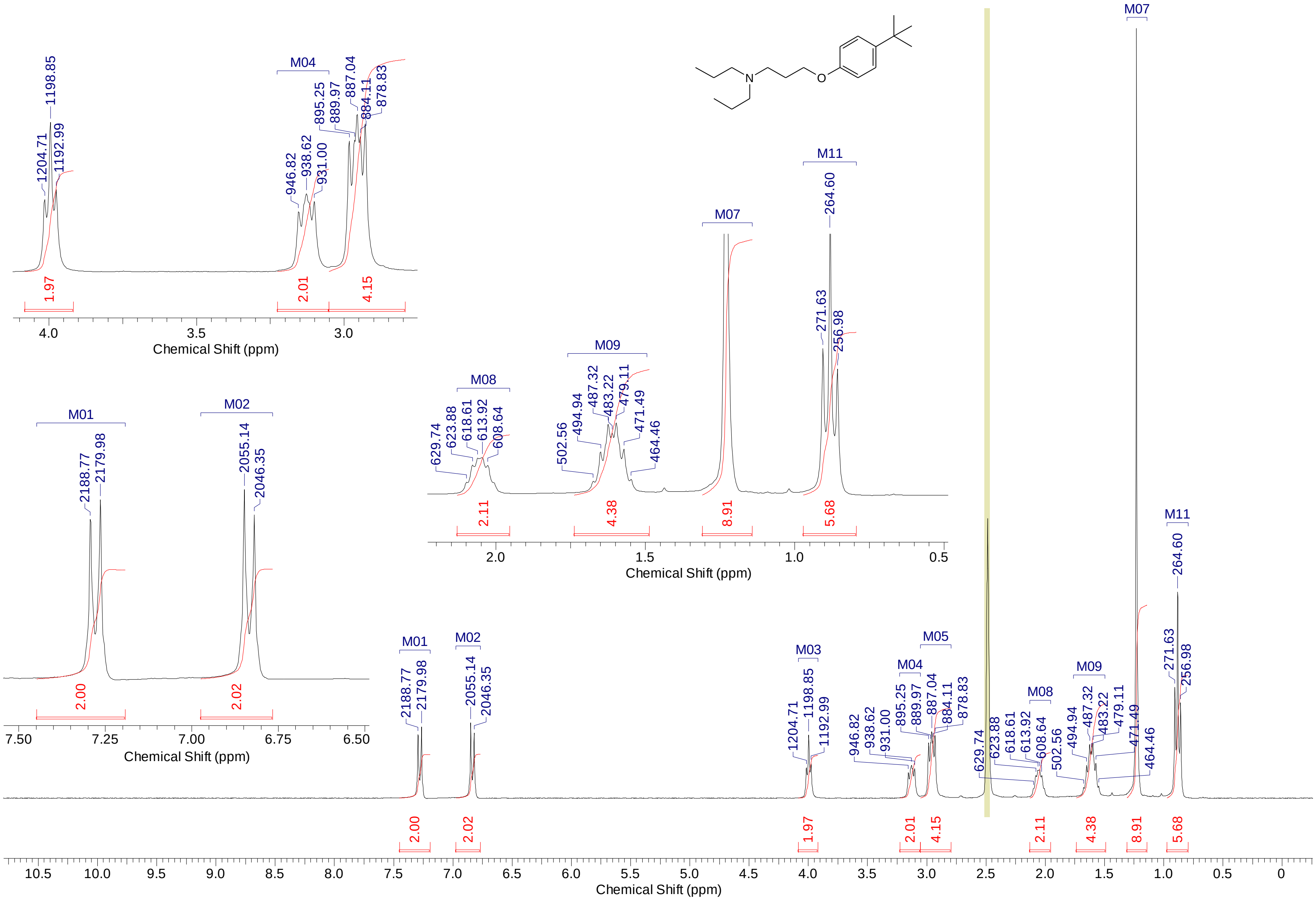

^{1}H NMR spectrum of 3-(4-(*tert*-butyl)phenoxy)-*N*,*N*-dipropylpropan-1-amine hydrogen oxalate (**9**)

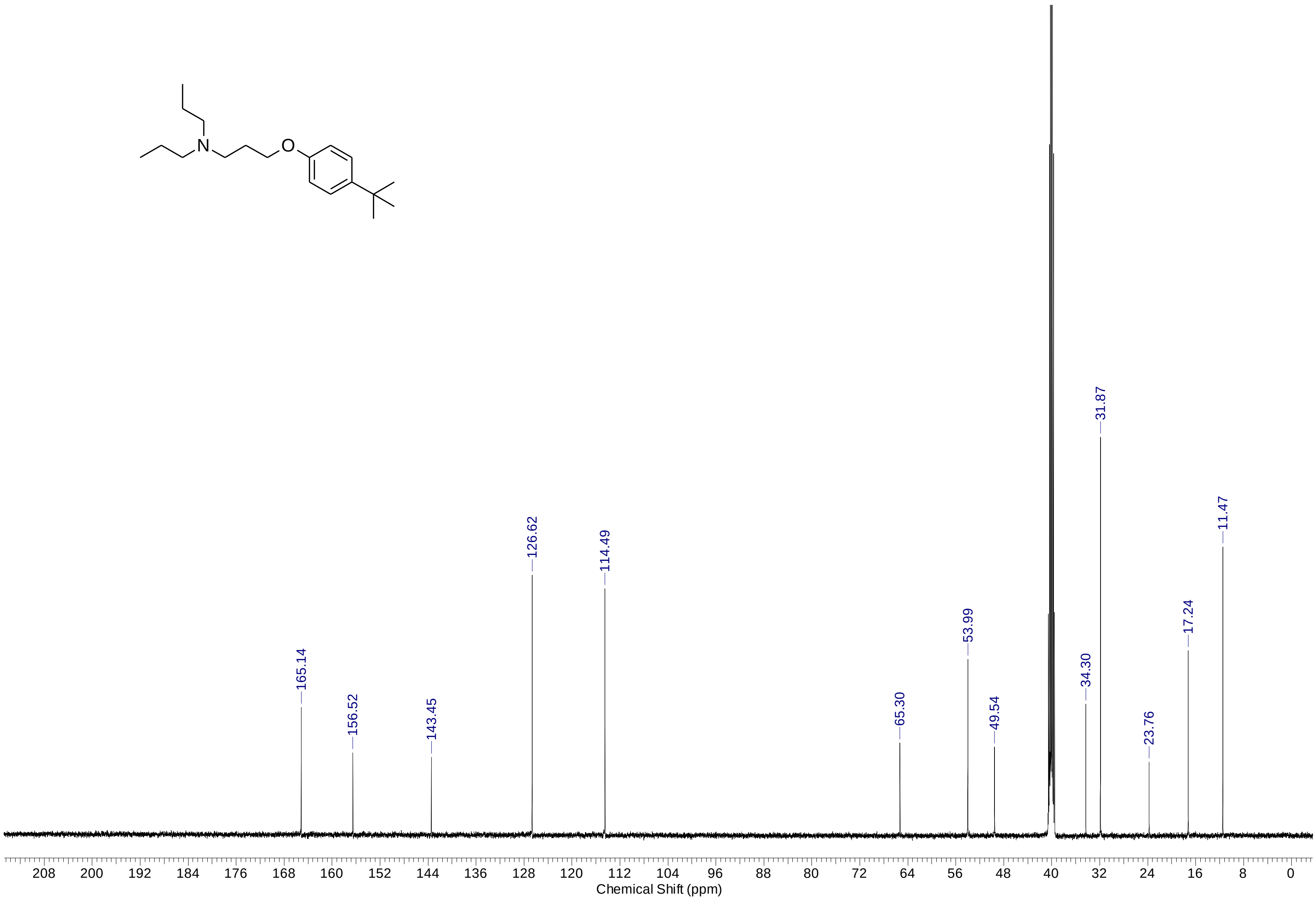

^{13}C NMR spectrum of 3-(4-(*tert*-butyl)phenoxy)-*N*,*N*-dipropylpropan-1-amine hydrogen oxalate (**9**)

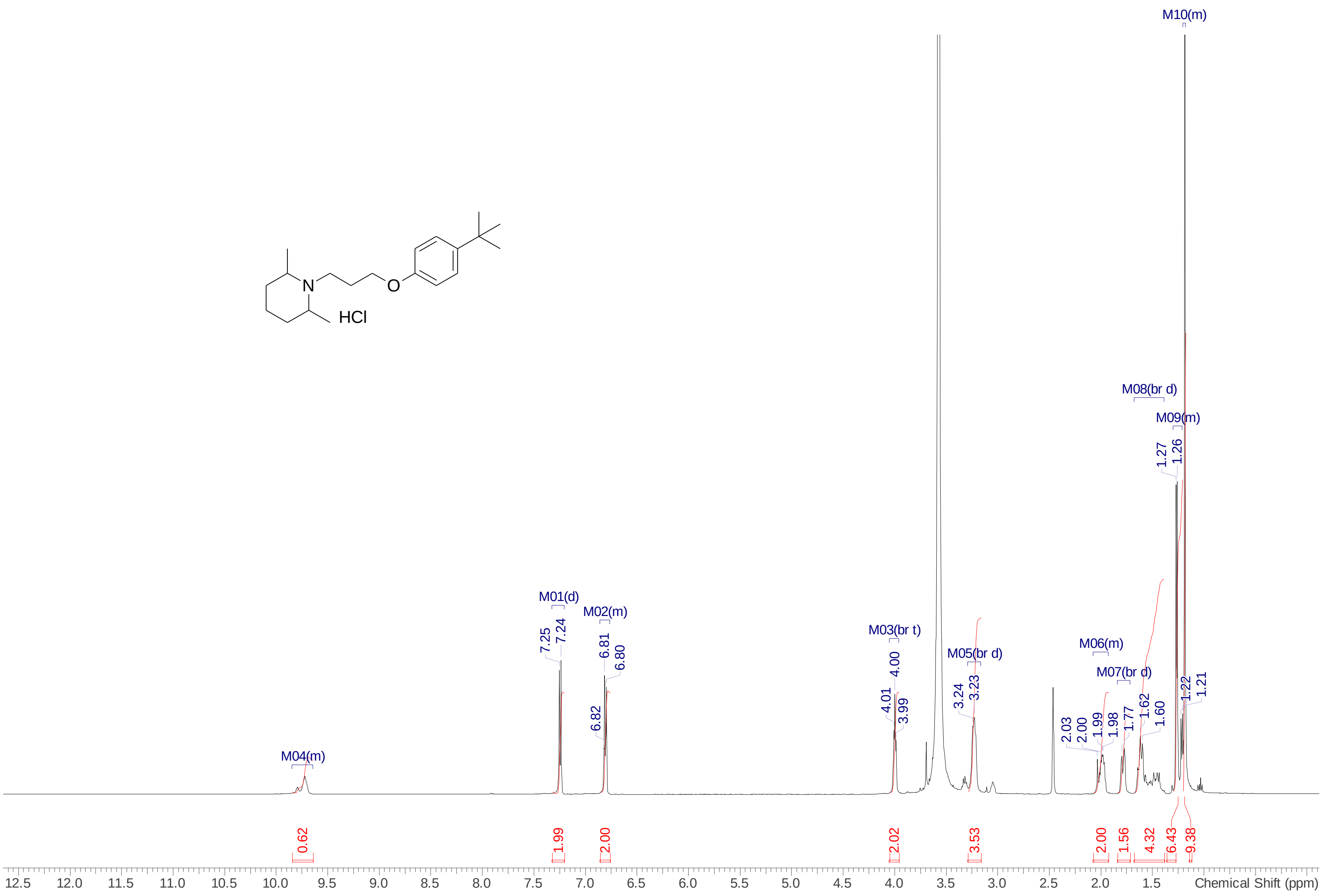

^{1}H NMR spectrum of 1-(3-(4-(*tert*-butyl)phenoxy)propyl)-2,6-dimethylpiperidine hydrogen chloride (**11**)

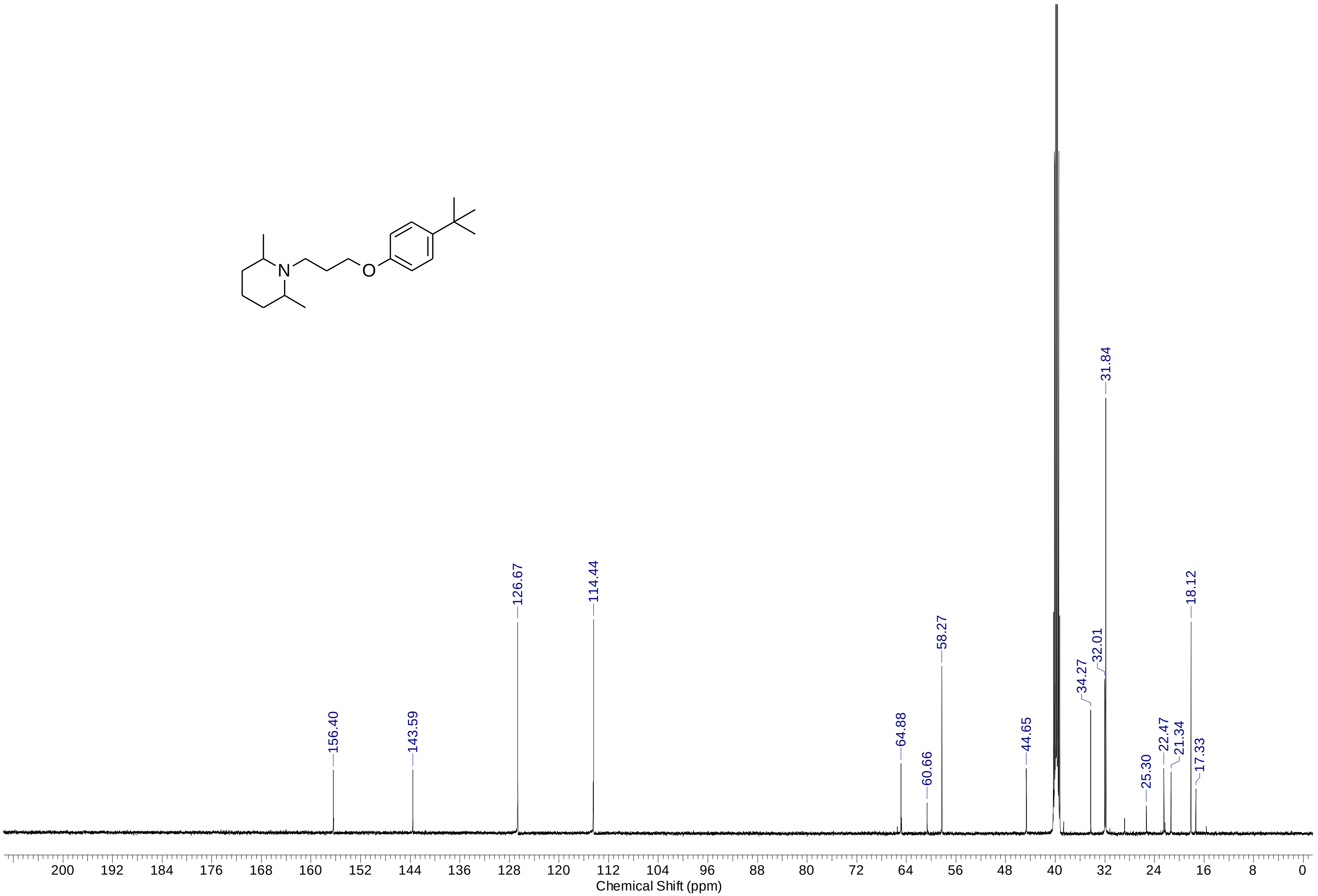

^{13}C NMR spectrum of 1-(3-(4-(*tert*-butyl)phenoxy)propyl)-2,6-dimethylpiperidine hydrogen chloride (**11**)

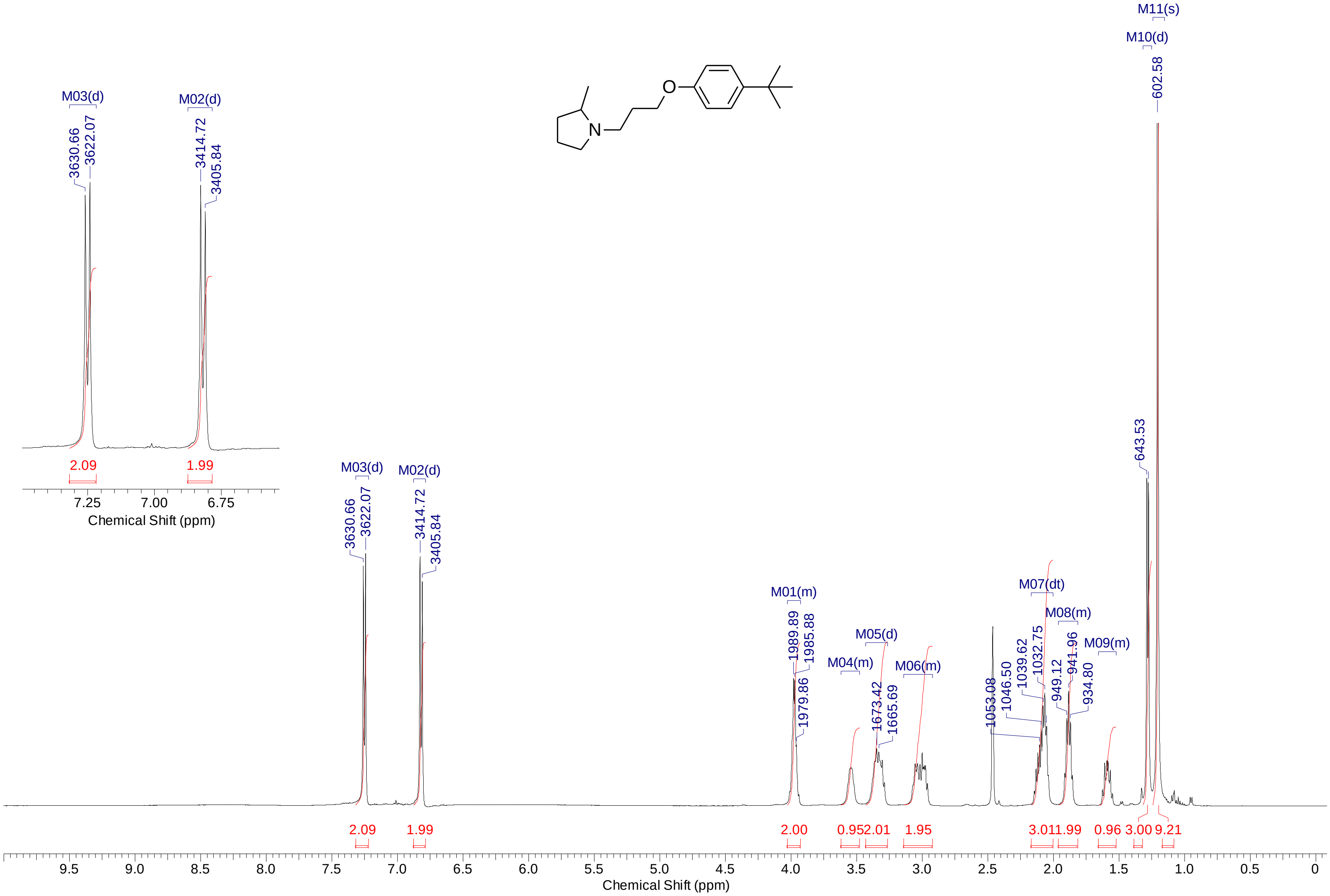

^{1}H NMR spectrum of 1-(3-(4-(*tert*-butyl)phenoxy)propyl)-2-methylpyrrolidine hydrogen oxalate (**13**)

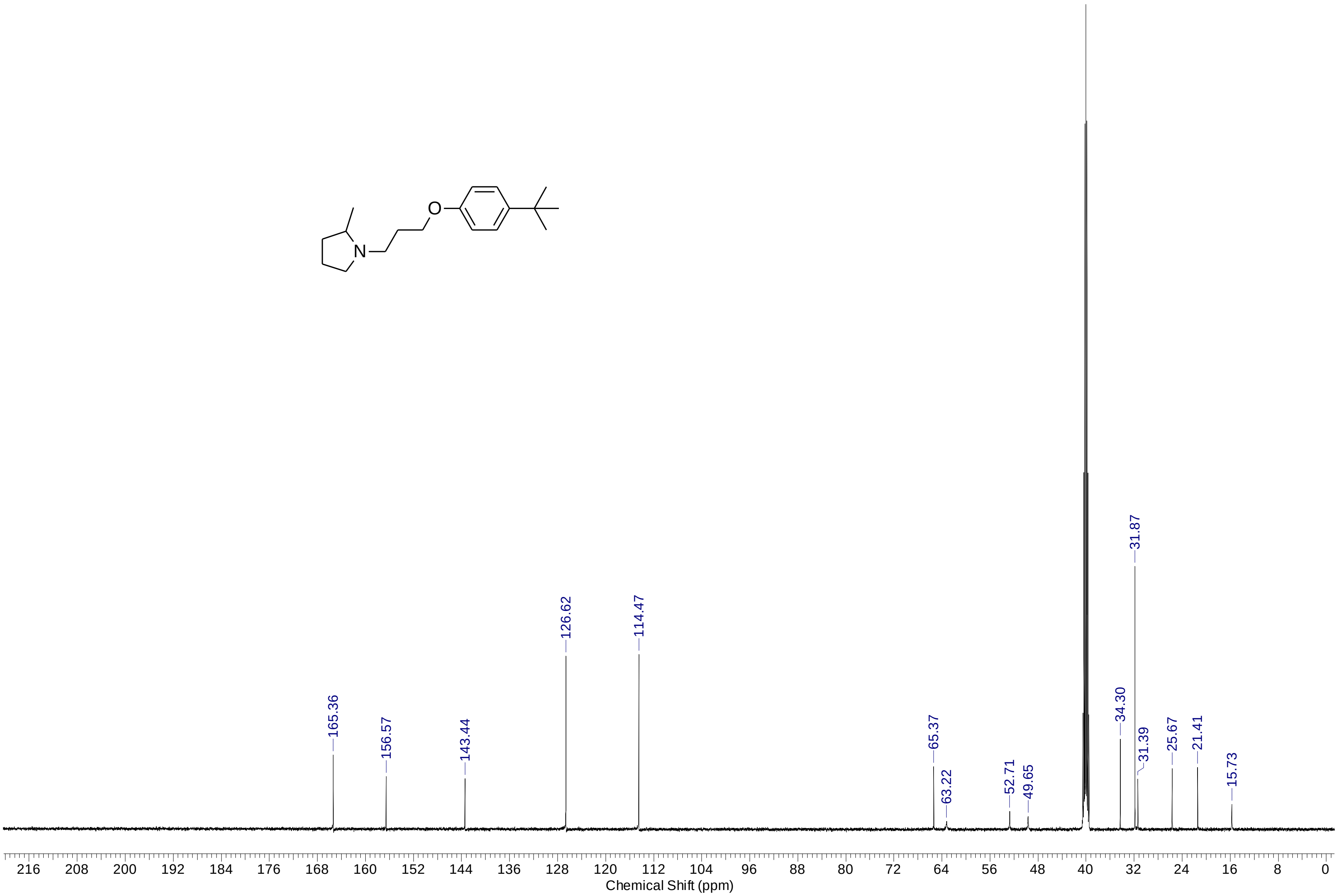

^{13}C NMR spectrum of 1-(3-(4-(*tert*-butyl)phenoxy)propyl)-2-methylpyrrolidine hydrogen oxalate (**13**)

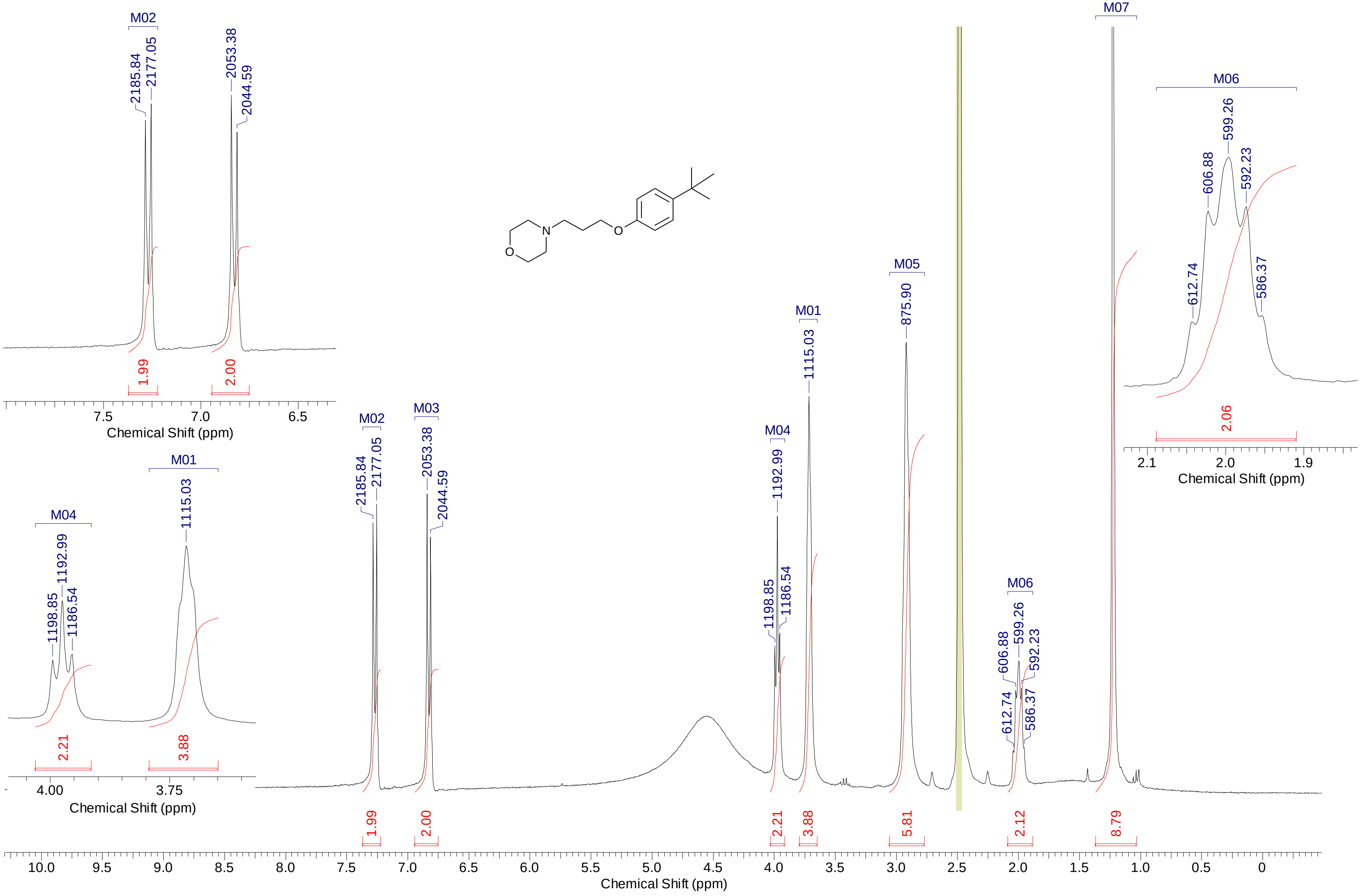

^{1}H NMR spectrum of 4-(3-(4-(*tert*-butyl)phenoxy)propyl)morpholine hydrogen oxalate (**14**)

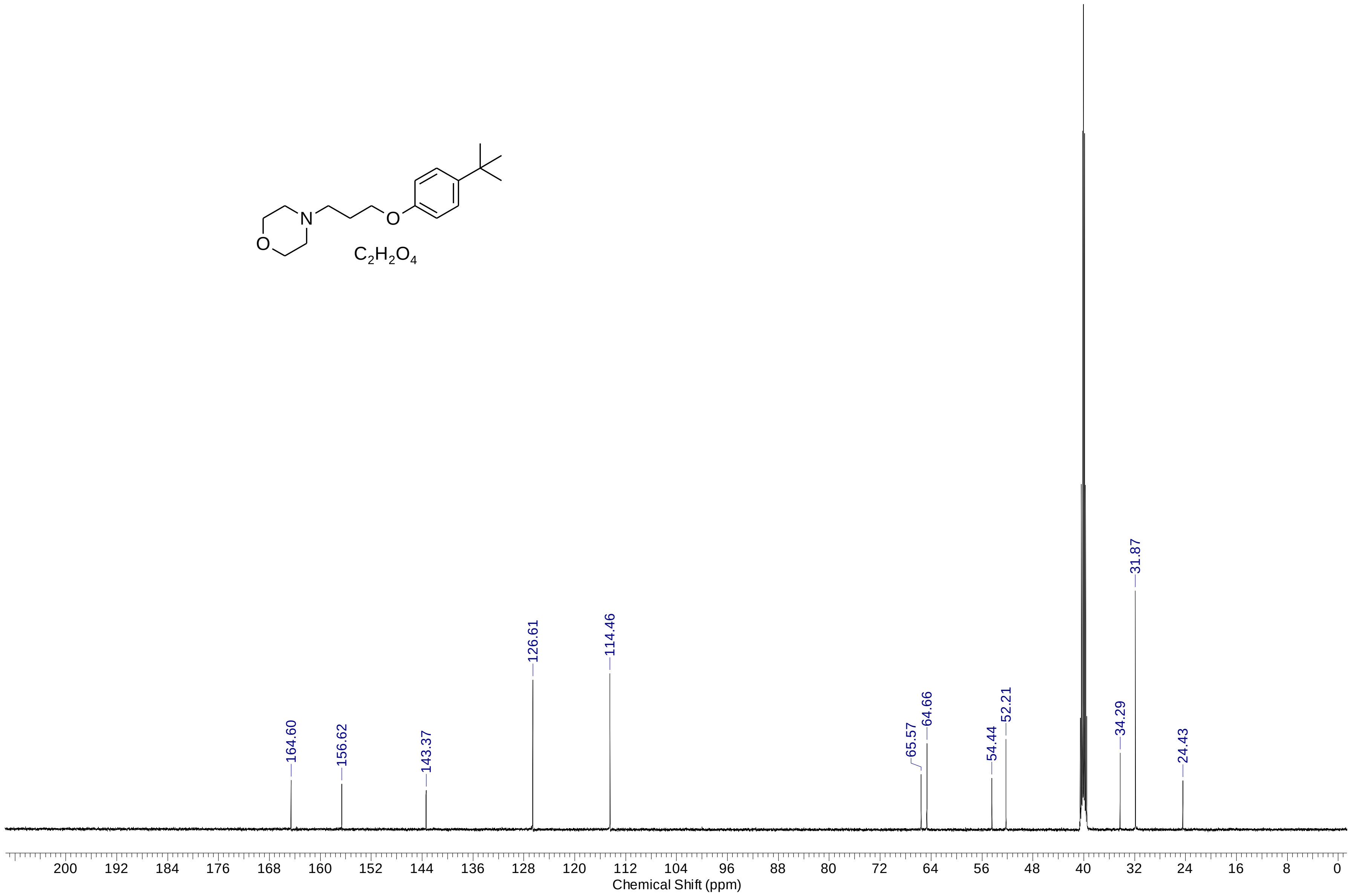

^{13}C NMR spectrum of 4-(3-(4-(*tert*-butyl)phenoxy)propyl)morpholine hydrogen oxalate (**14**)

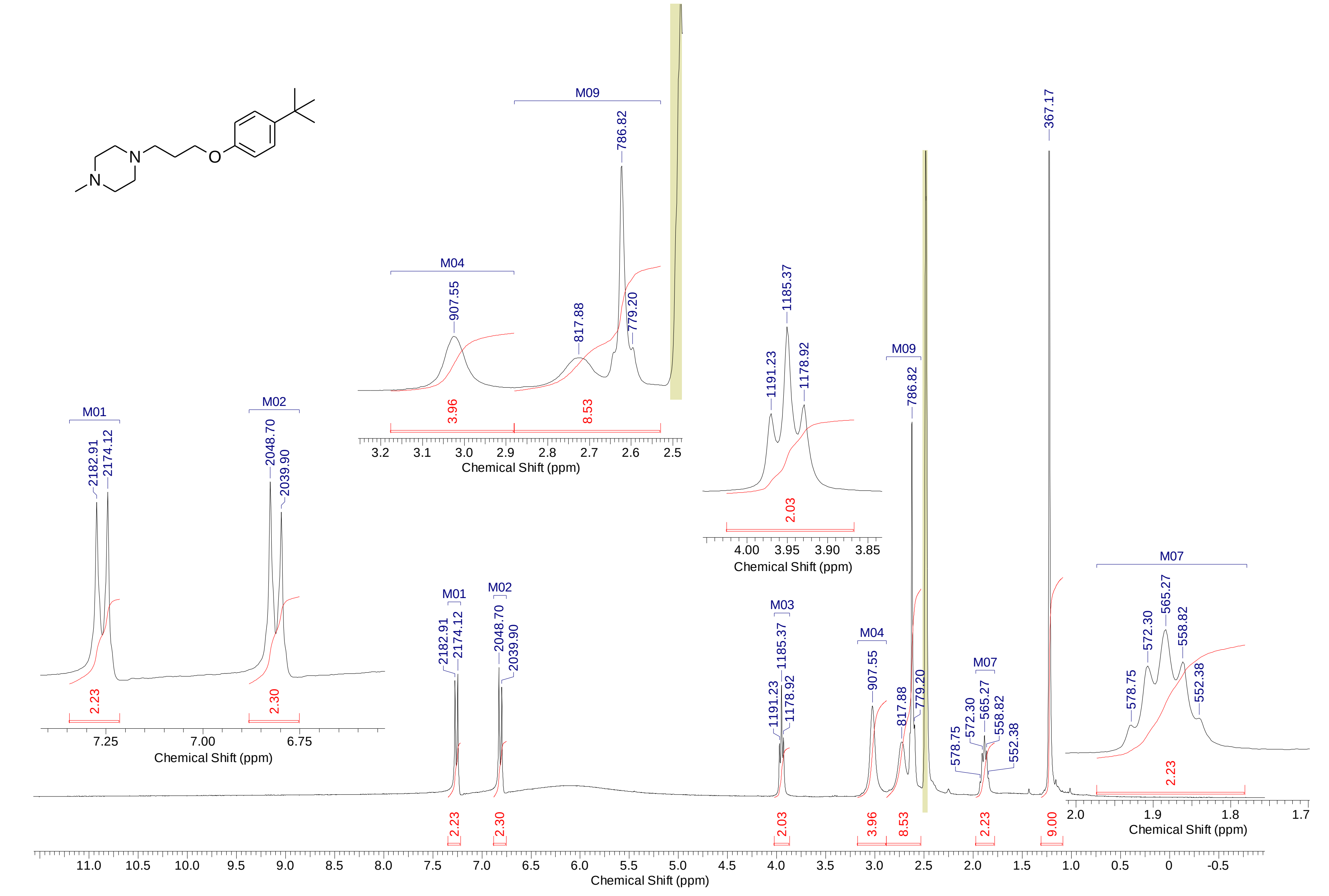

^{1}H NMR spectrum of 1-(3-(4-(*tert*-butyl)phenoxy)propyl)-4-methylpiperazine hydrogen oxalate (**15**)

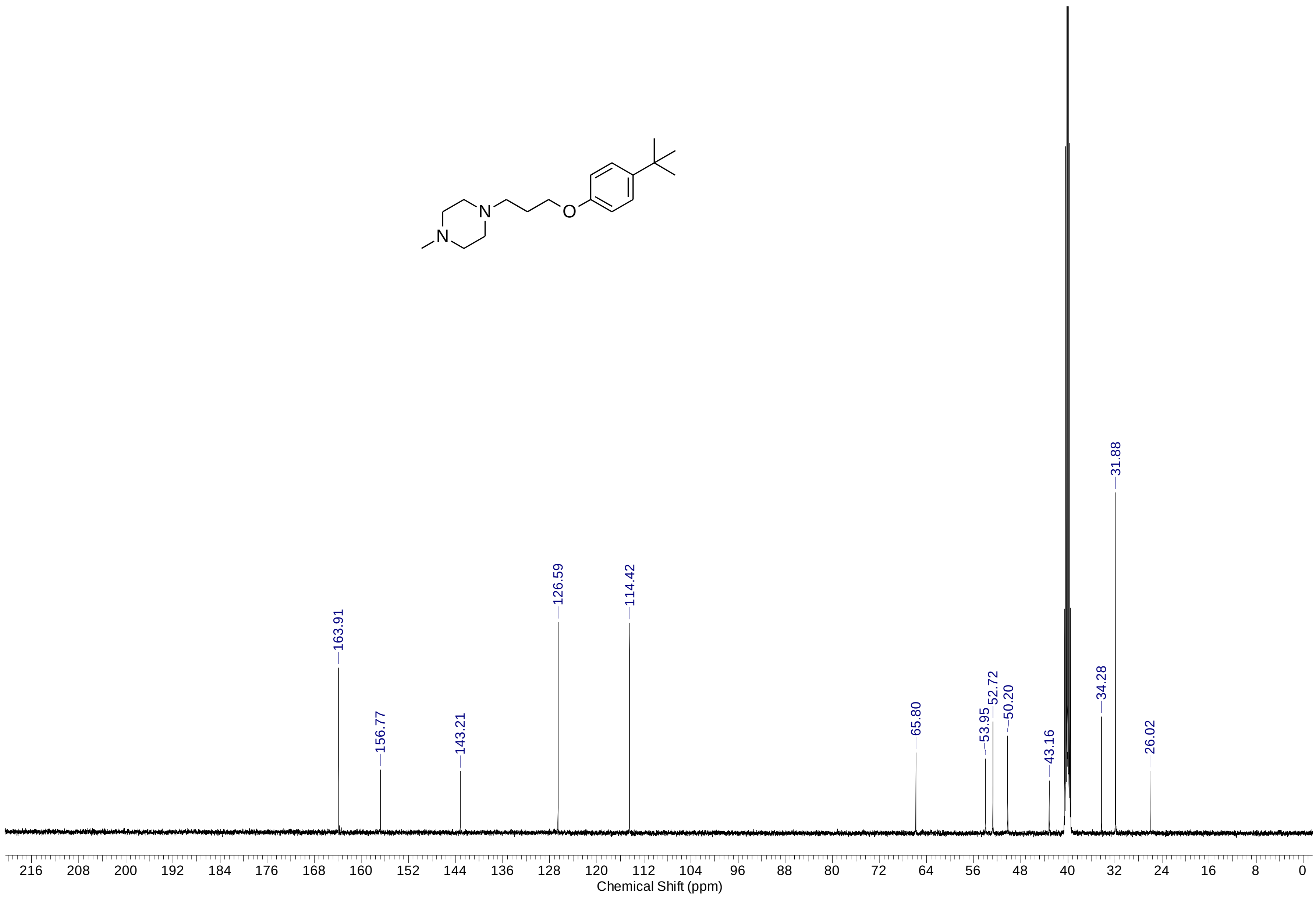

^{13}C NMR spectrum of 1-(3-(4-(*tert*-butyl)phenoxy)propyl)-4-methylpiperazine hydrogen oxalate (**15**)

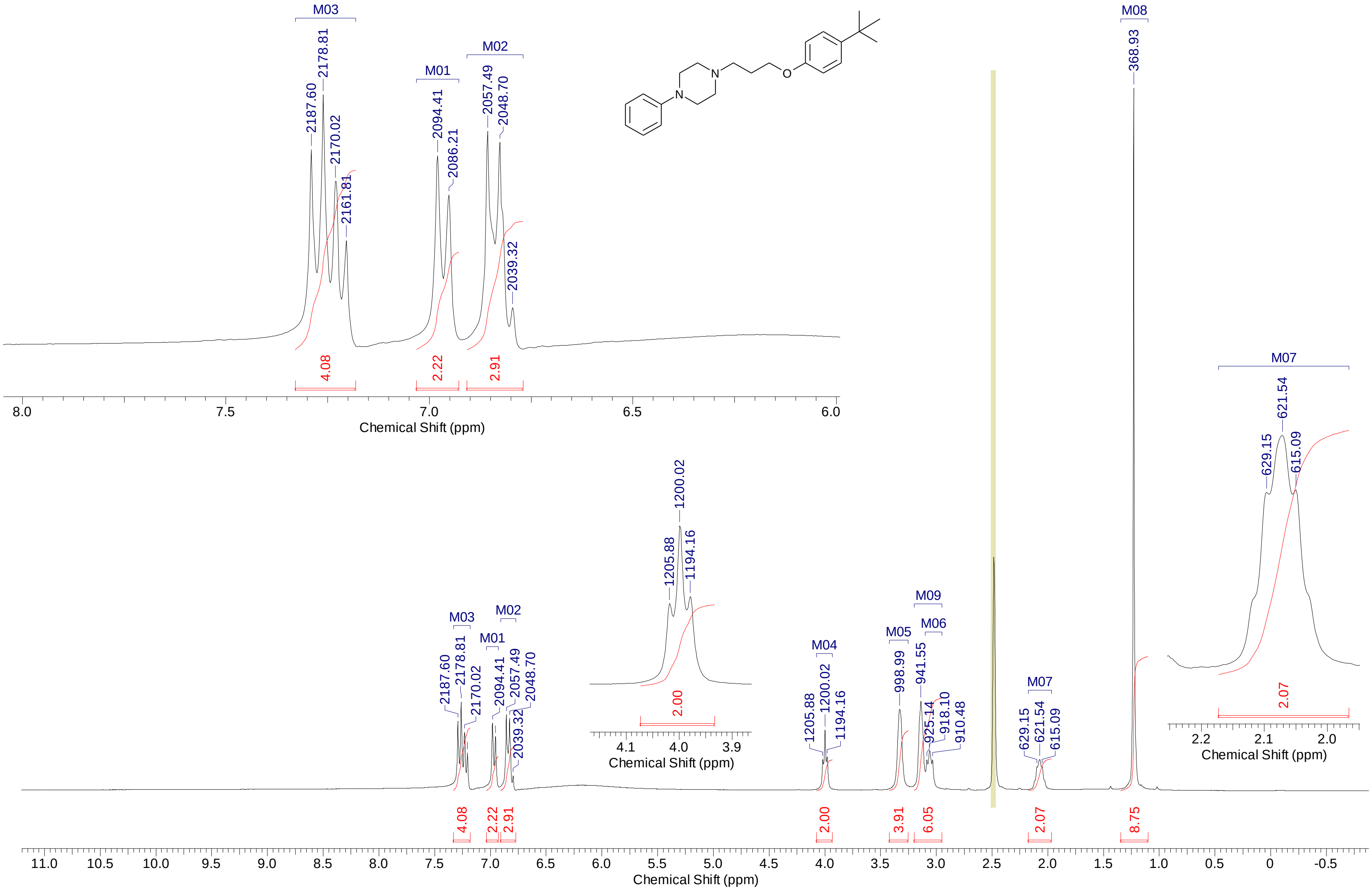

^{1}H NMR spectrum of 1-(3-(4-(*tert*-butyl)phenoxy)propyl)-4-phenylpiperazine hydrogen oxalate (**16**)

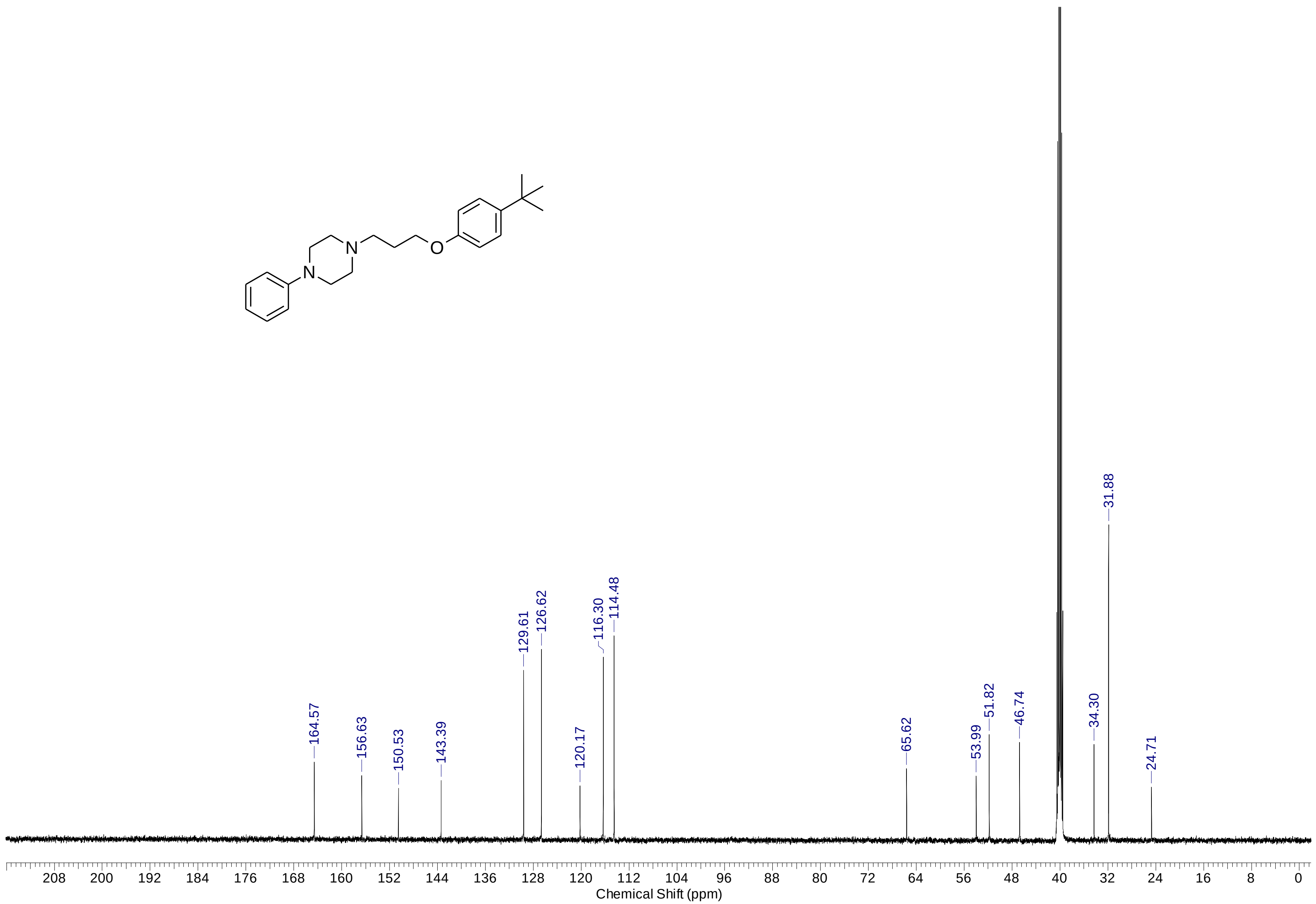

^{13}C NMR spectrum of 1-(3-(4-(*tert*-butyl)phenoxy)propyl)-4-phenylpiperazine hydrogen oxalate (**16**)

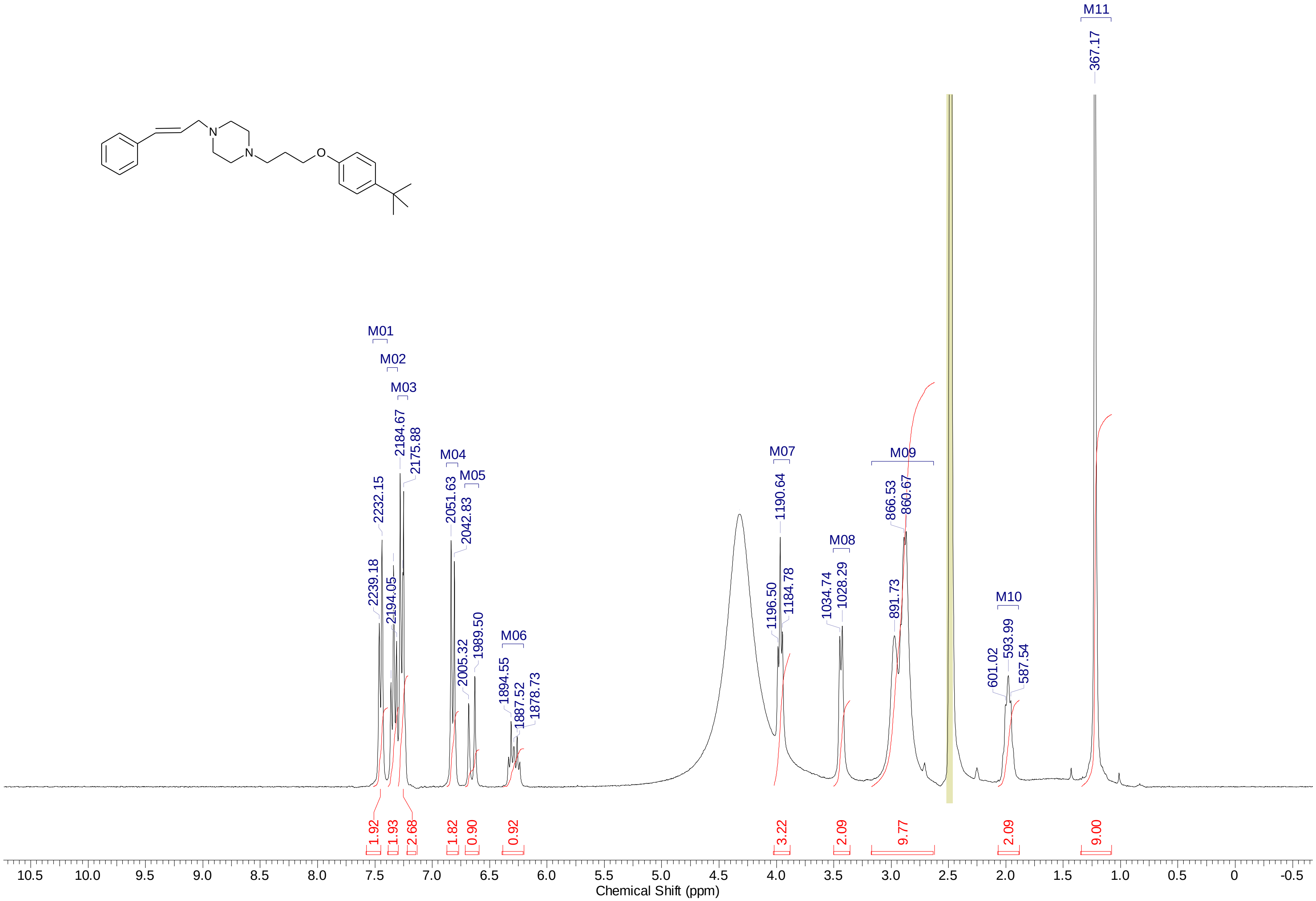

^{1}H NMR spectrum of 1-(3-(4-(*tert*-butyl)phenoxy)propyl)-4-cinnamylpiperazine hydrogen oxalate (**17**)

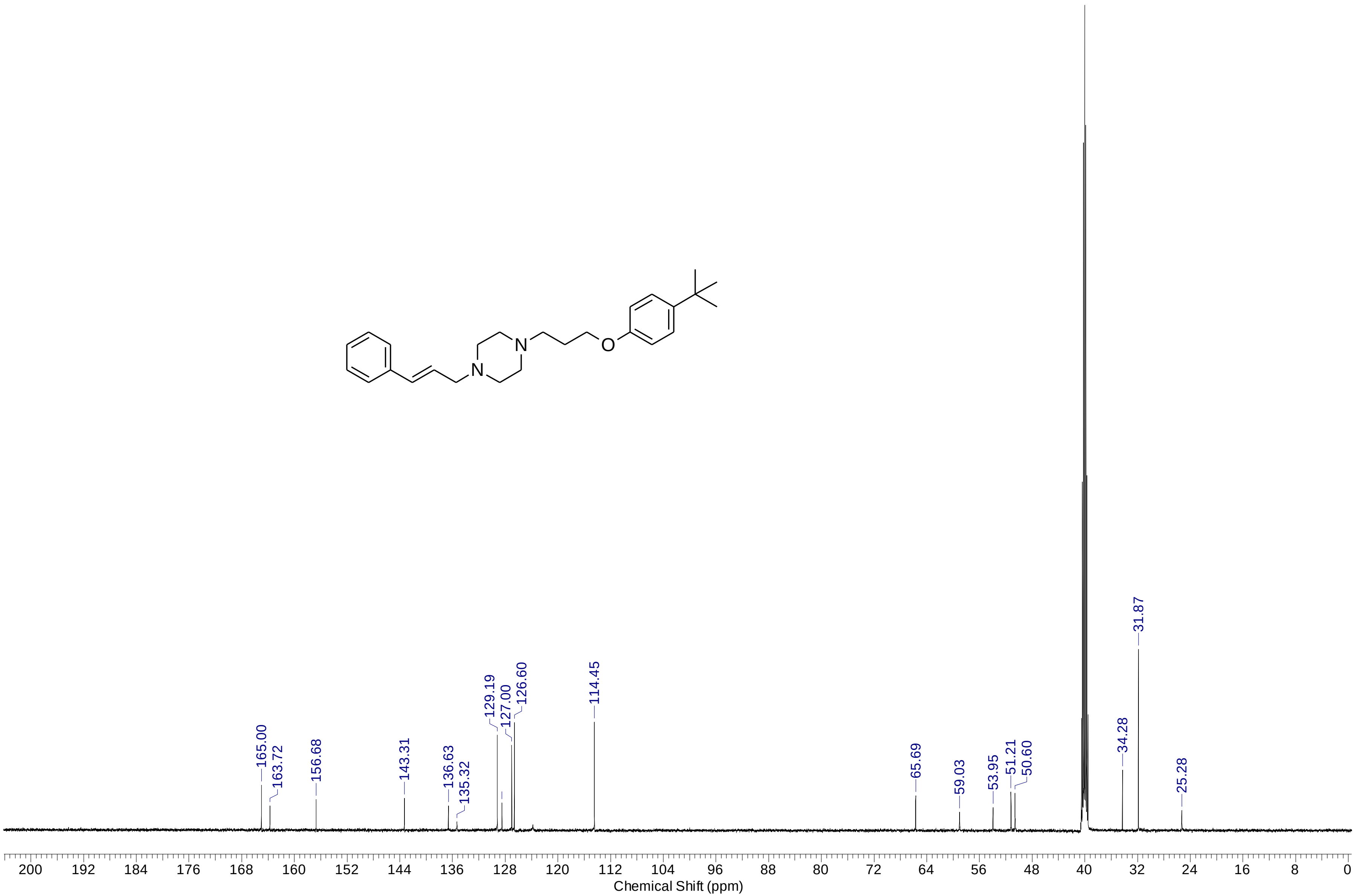

^{13}C NMR spectrum of 1-(3-(4-(*tert*-butyl)phenoxy)propyl)-4-cinnamylpiperazine hydrogen oxalate (**17**)

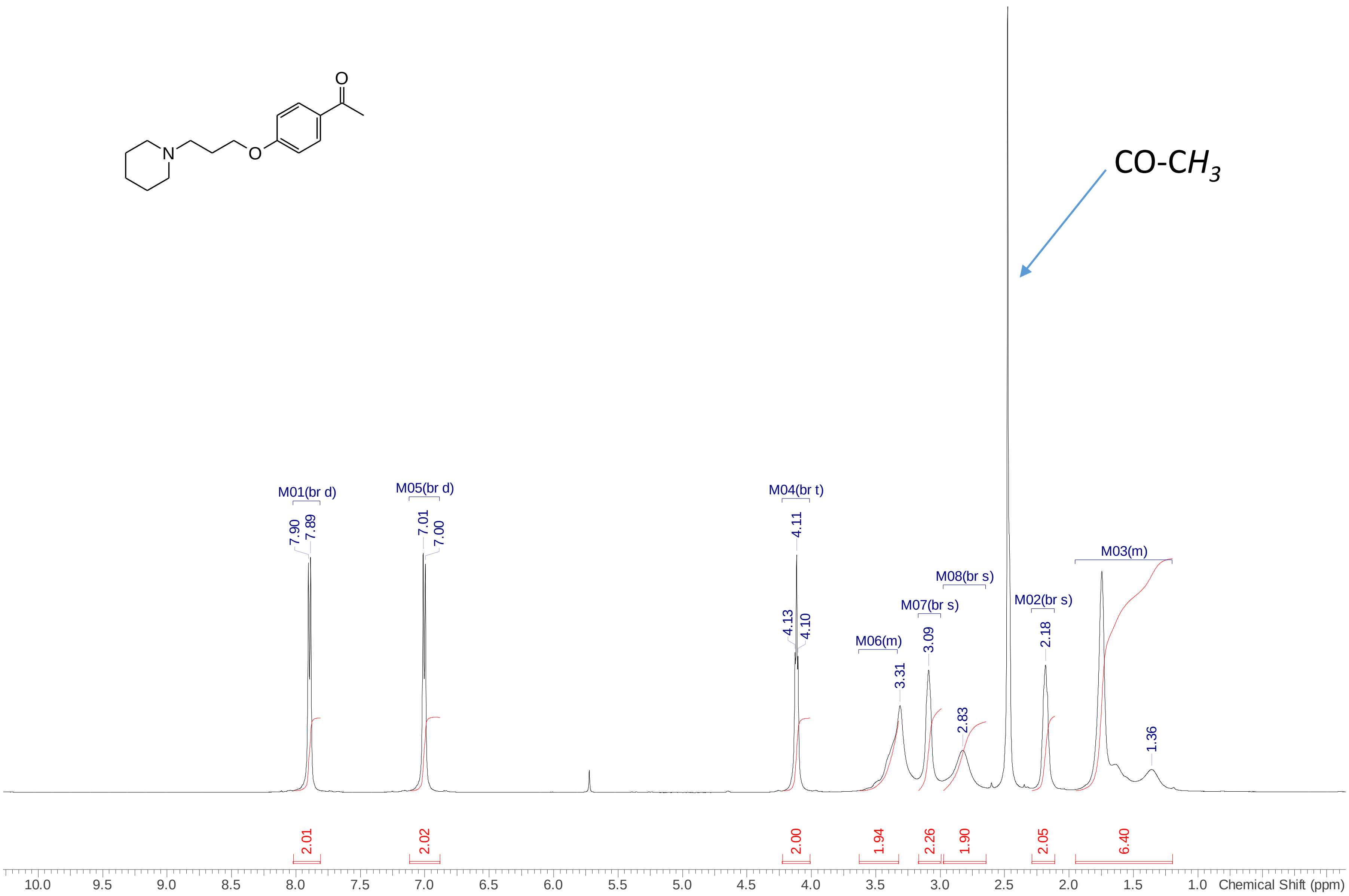

^{1}H NMR spectrum of 1-(4-(3-(piperidin-1-yl)propoxy)phenyl)ethan-1-one hydrogen oxalate (**18**)

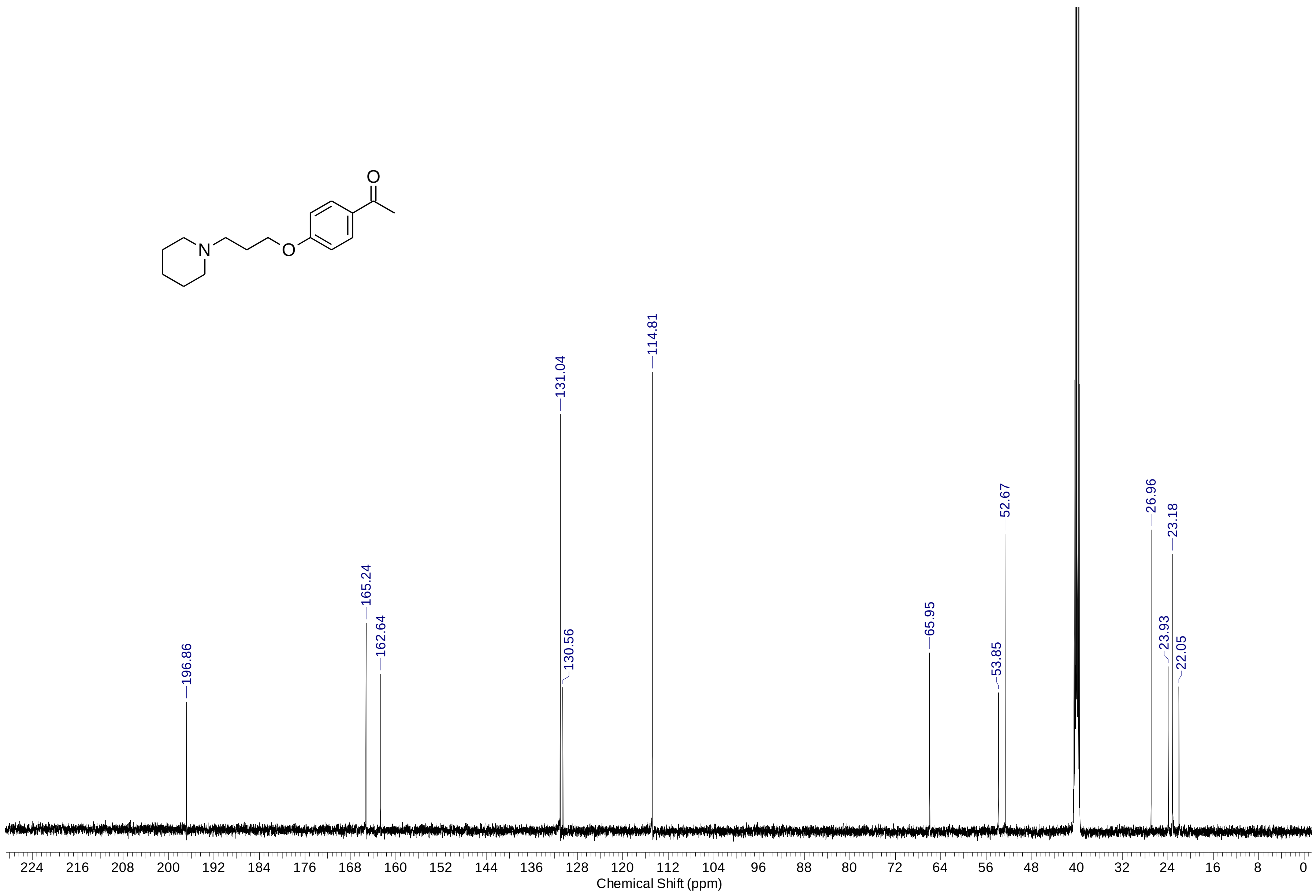

^{13}C NMR spectrum of 1-(4-(3-(piperidin-1-yl)propoxy)phenyl)ethan-1-one hydrogen oxalate (**18**)

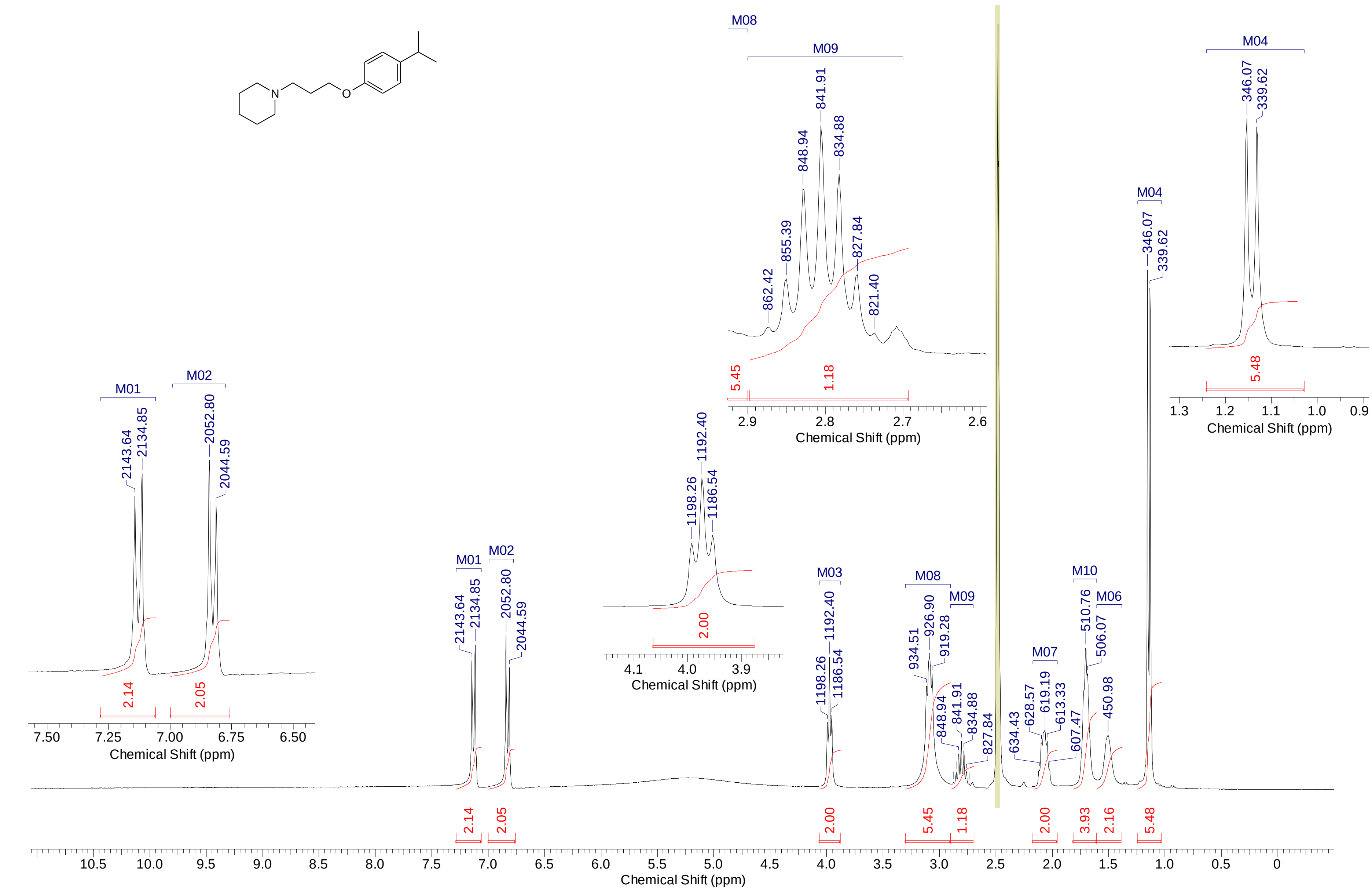

^{1}H NMR spectrum of 1-(3-(4-isopropylphenoxy)propyl)piperidine hydrogen oxalate (**19**)

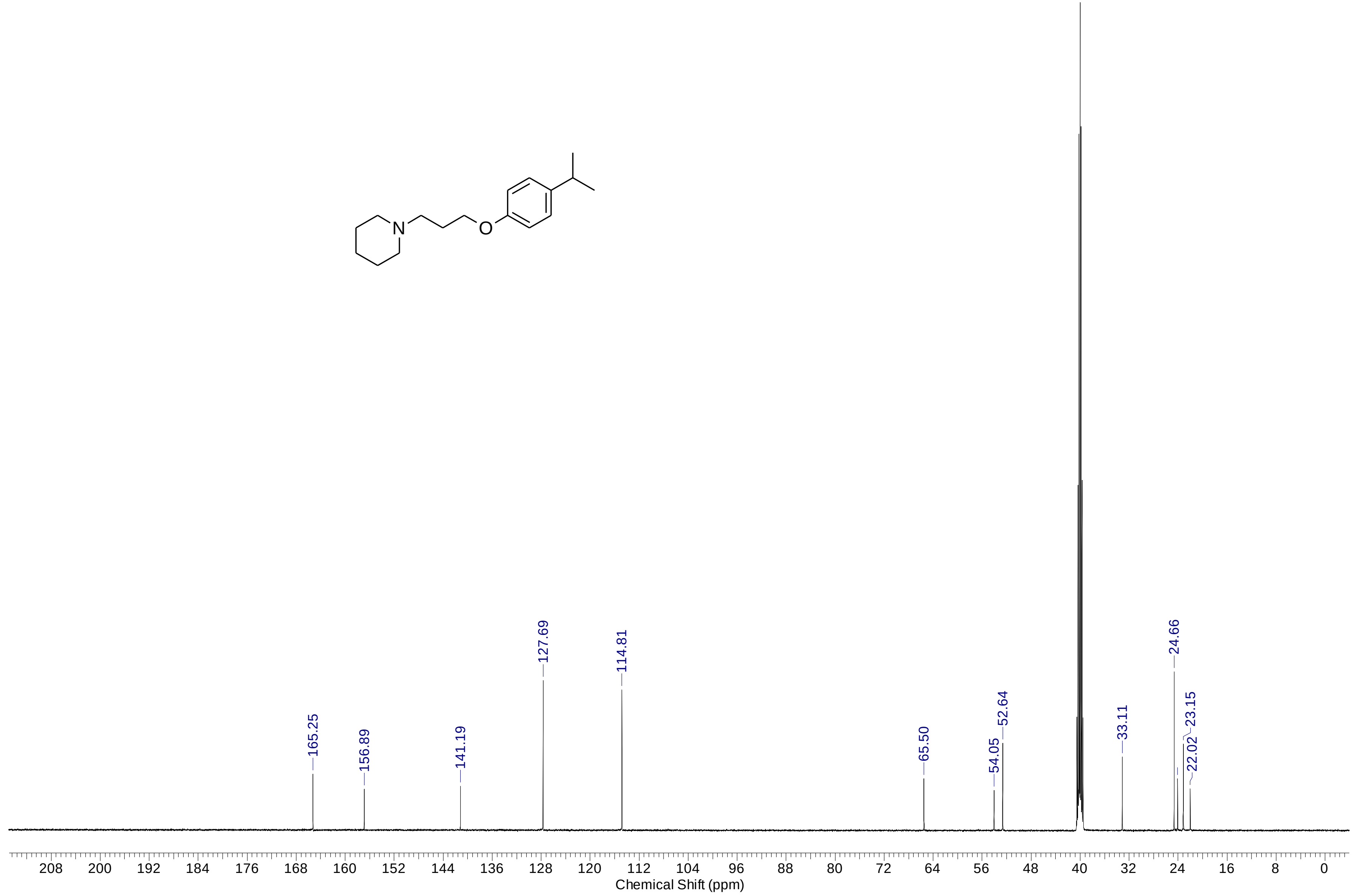

^{13}C NMR spectrum of 1-(3-(4-isopropylphenoxy)propyl)piperidine hydrogen oxalate (**19**)

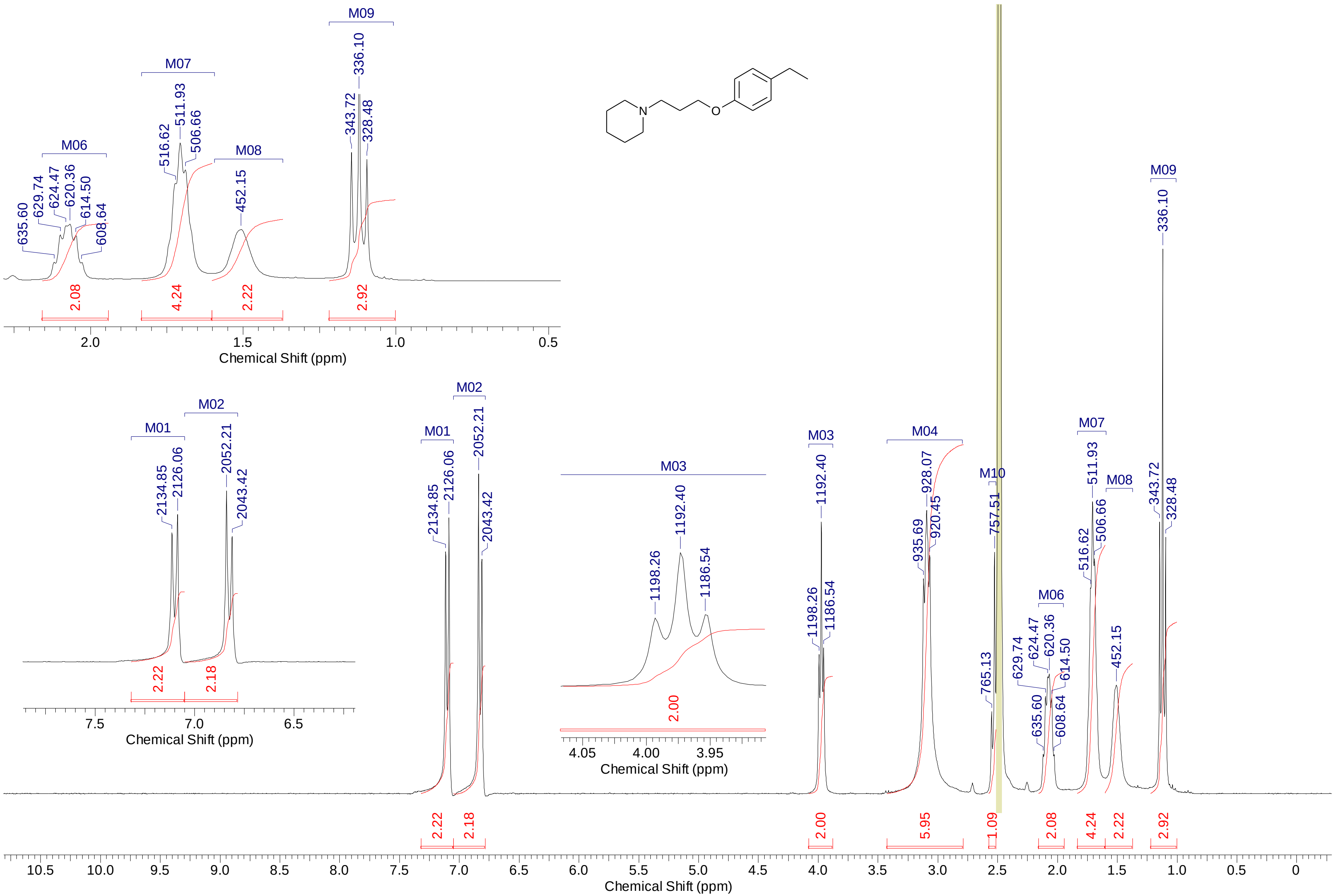

^{1}H NMR spectrum of 1-(3-(4-ethylphenoxy)propyl)piperidine hydrogen oxalate (**20**)

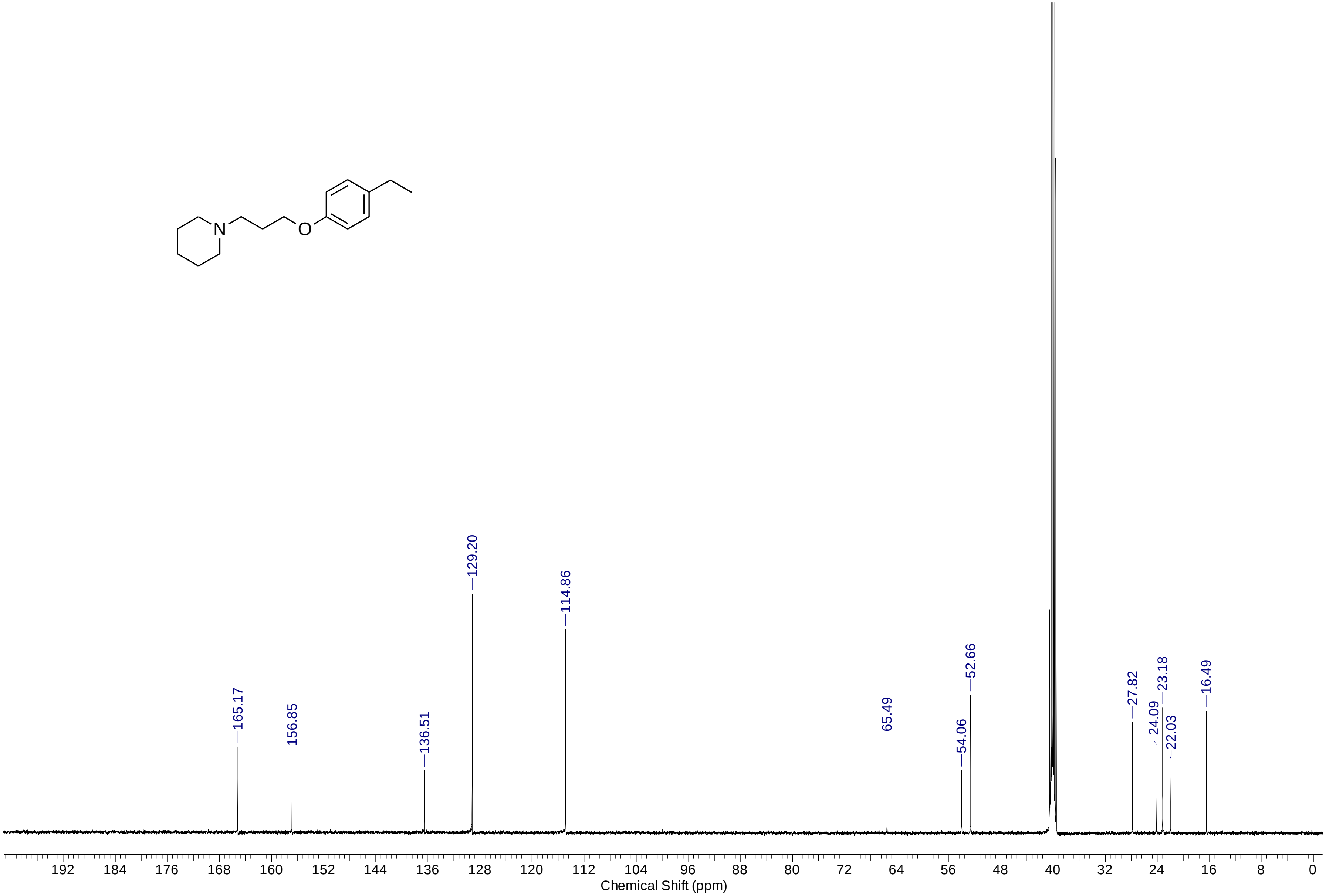

^{13}C NMR spectrum of 1-(3-(4-ethylphenoxy)propyl)piperidine hydrogen oxalate (**20**)

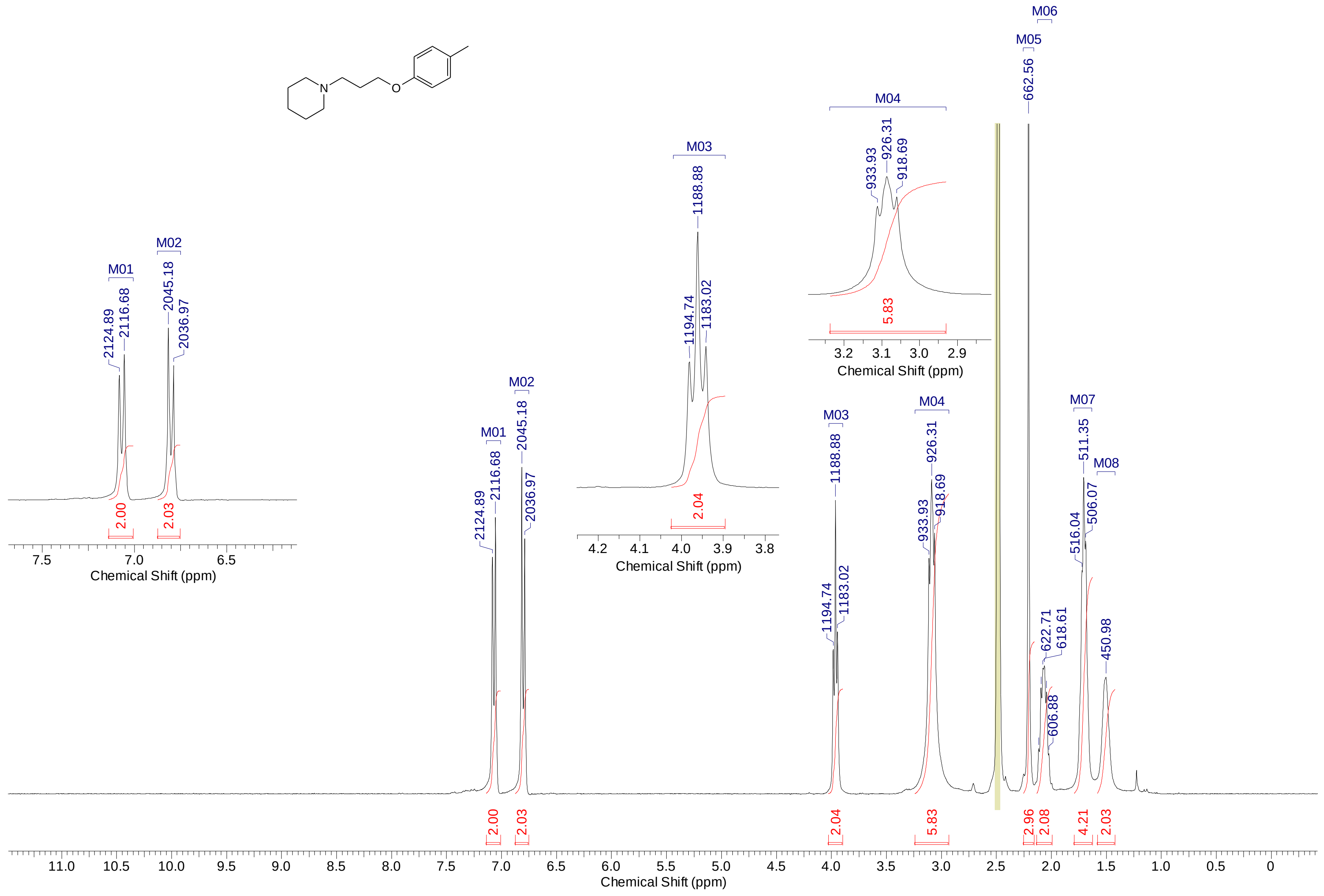

^{1}H NMR spectrum of 1-(3-(*p*-tolyloxy)propyl)piperidine hydrogen oxalate (**21**)

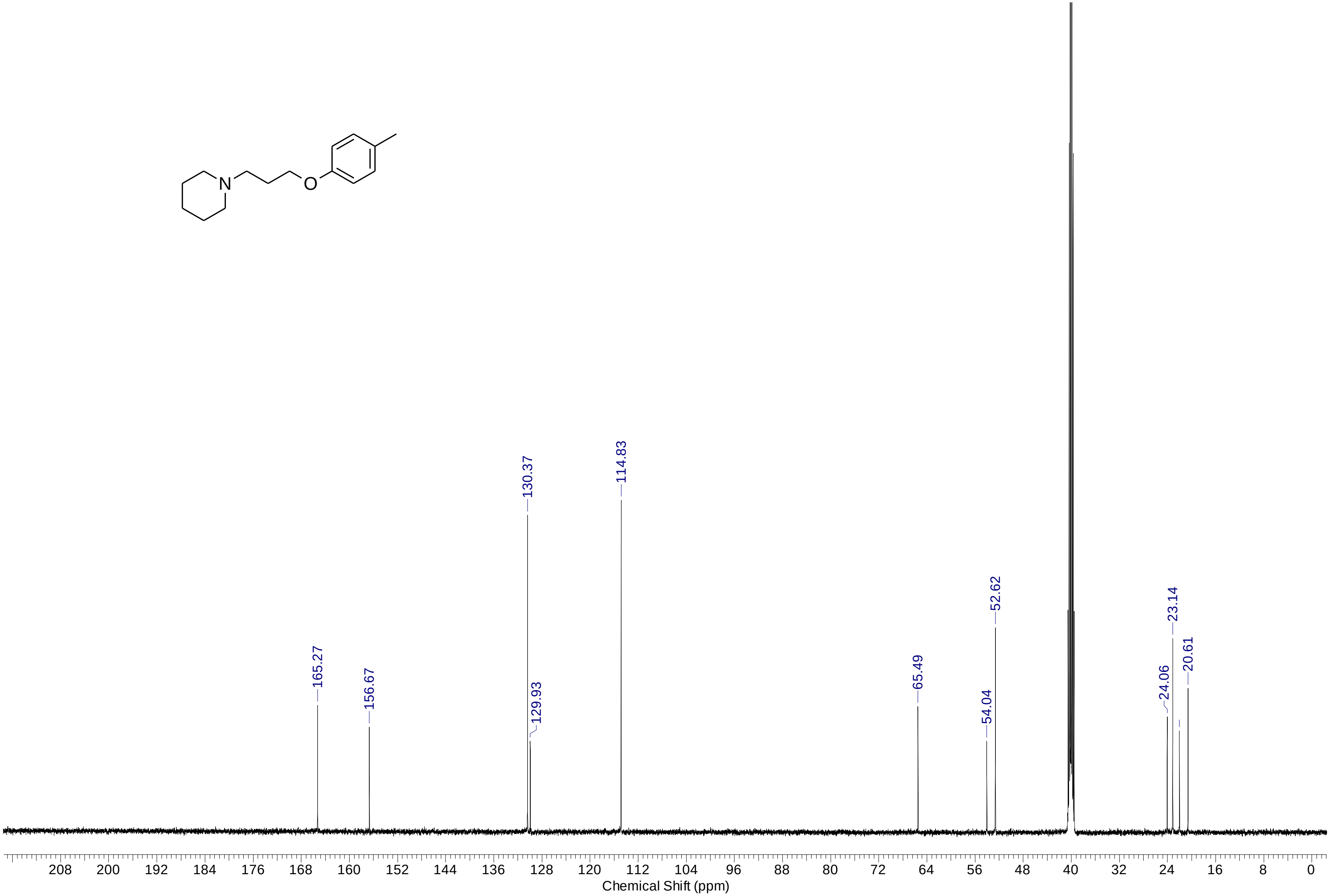

^{13}C NMR spectrum of 1-(3-(*p*-tolyloxy)propyl)piperidine hydrogen oxalate (**21**)

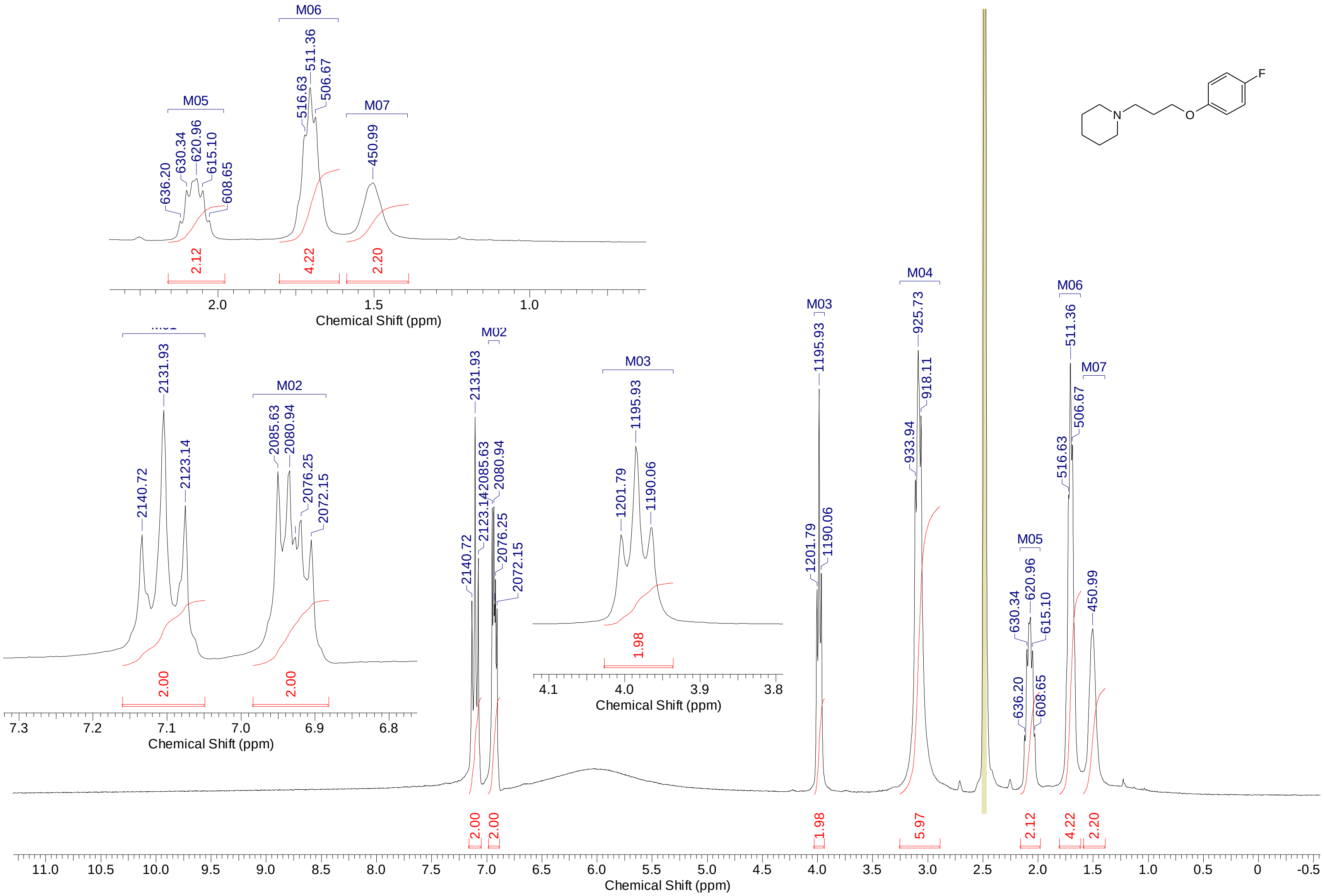

^{1}H NMR spectrum of 1-(3-(4-fluorophenoxy)propyl)piperidine hydrogen oxalate (**22**)

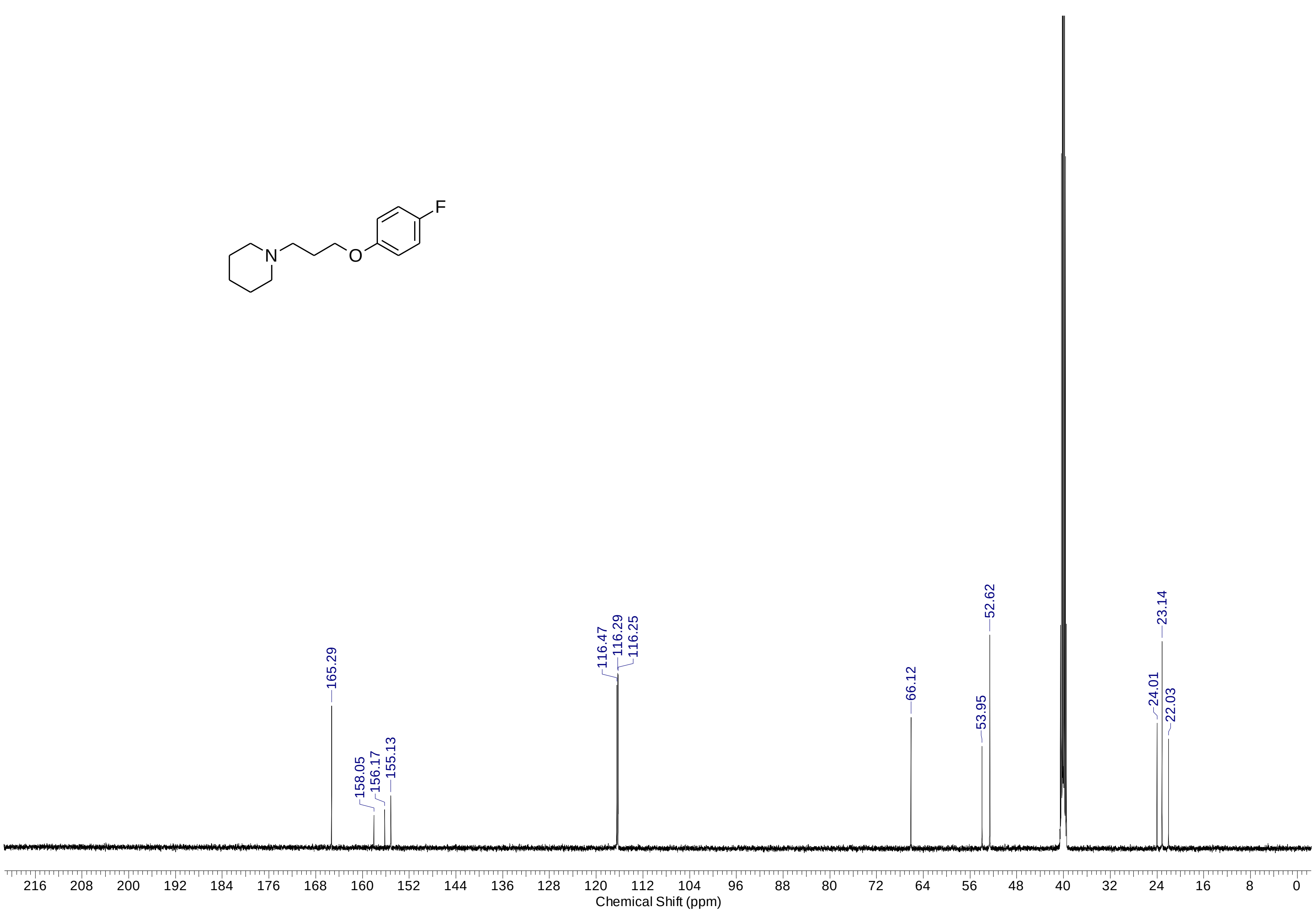

^{13}C NMR spectrum of 1-(3-(4-fluorophenoxy)propyl)piperidine hydrogen oxalate (**22**)

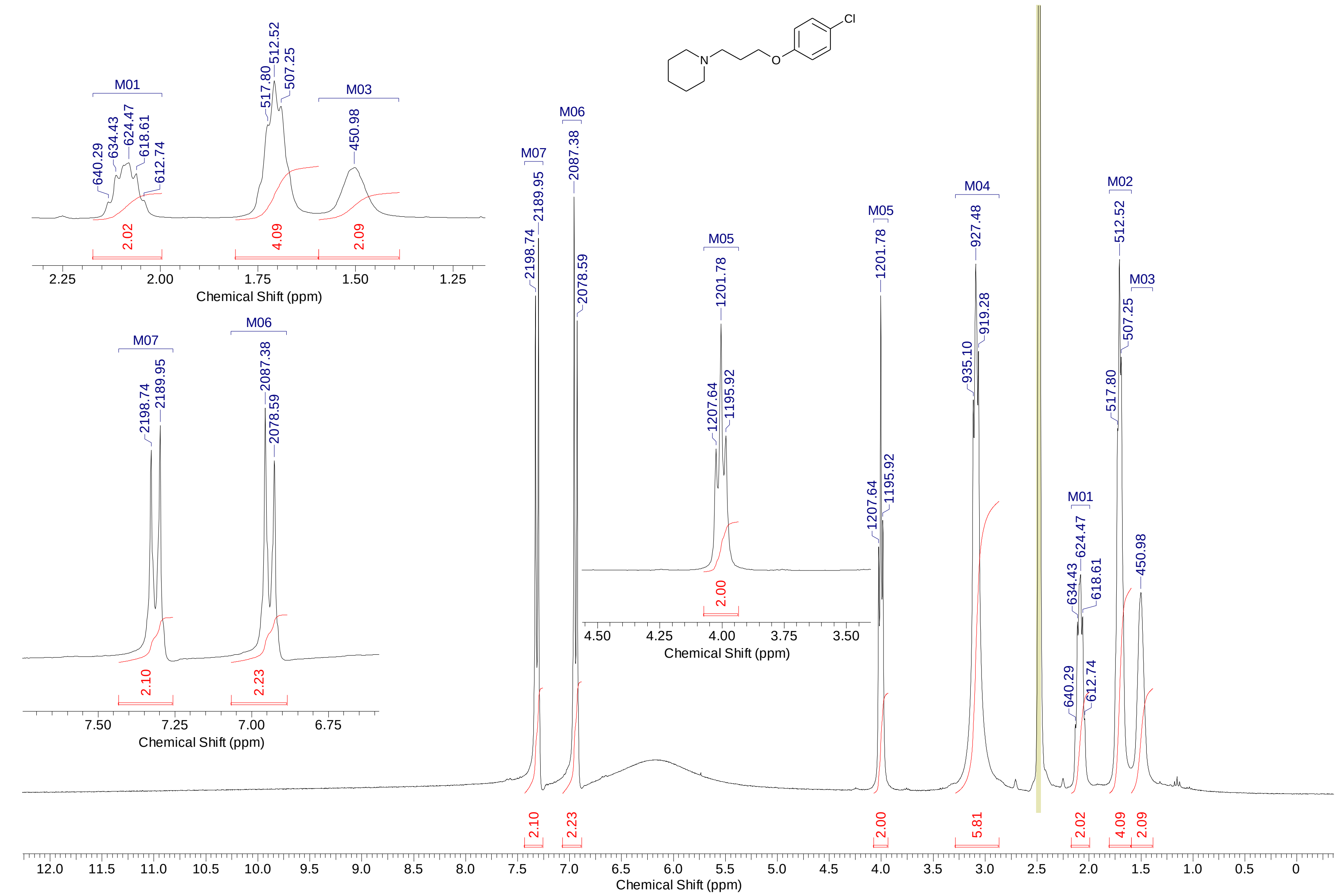

^{1}H NMR spectrum of 1-(3-(4-chlorophenoxy)propyl)piperidine hydrogen oxalate (**23**)

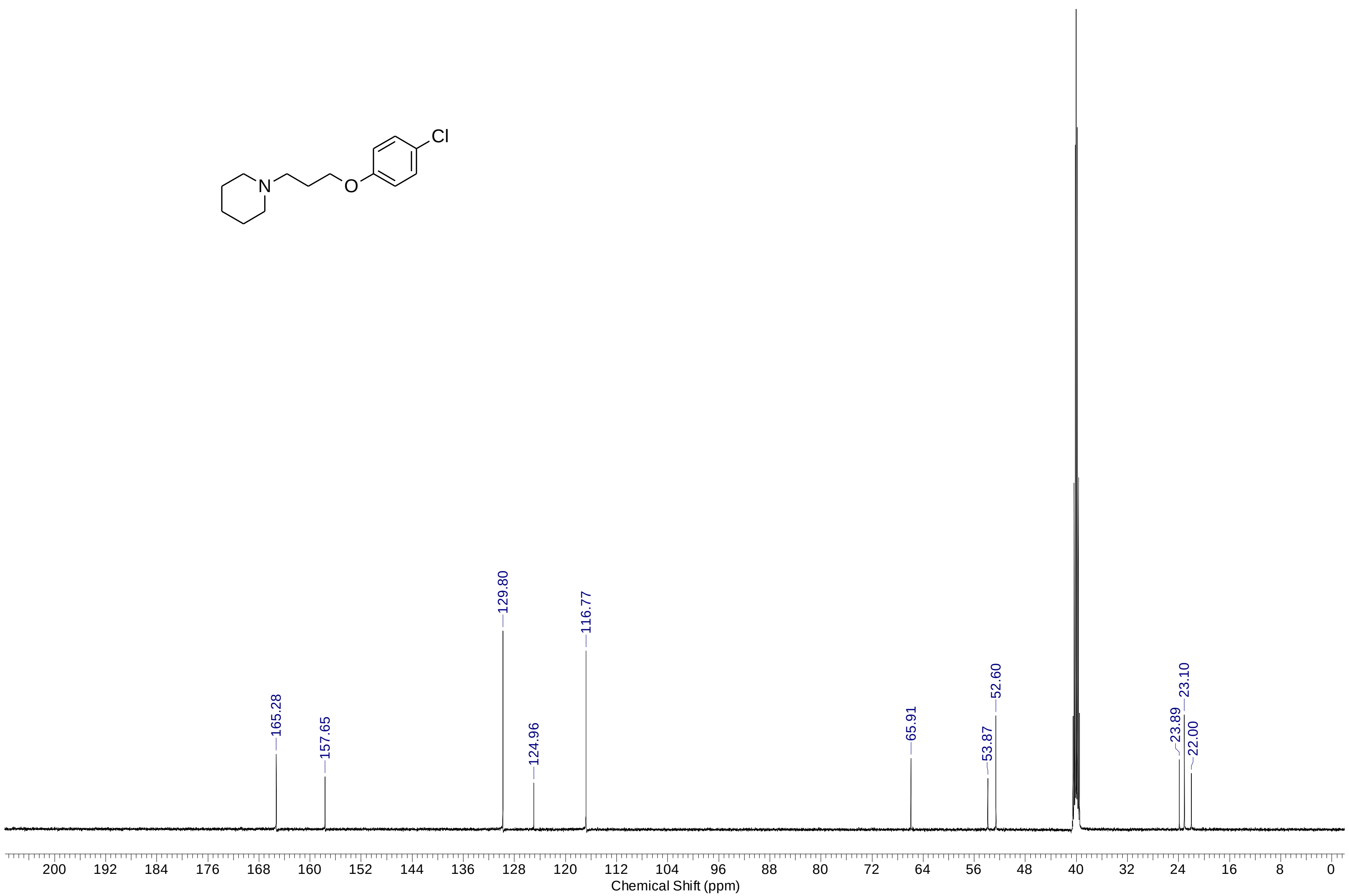

^{13}C NMR spectrum of 1-(3-(4-chlorophenoxy)propyl)piperidine hydrogen oxalate (**23**)

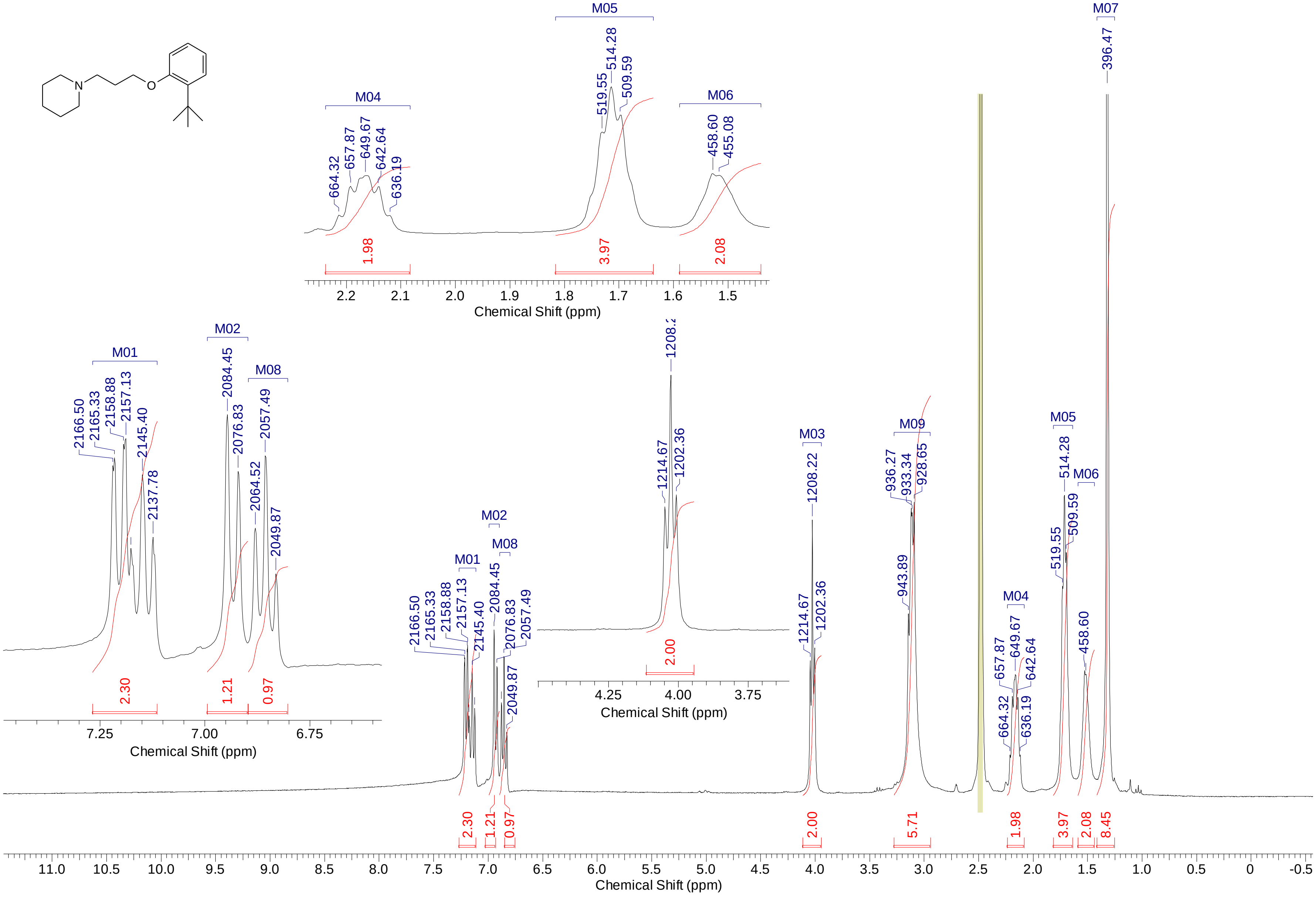

^{1}H NMR spectrum of 1-(3-(2-(*tert*-butyl)phenoxy)propyl)piperidine hydrogen oxalate (**24**)

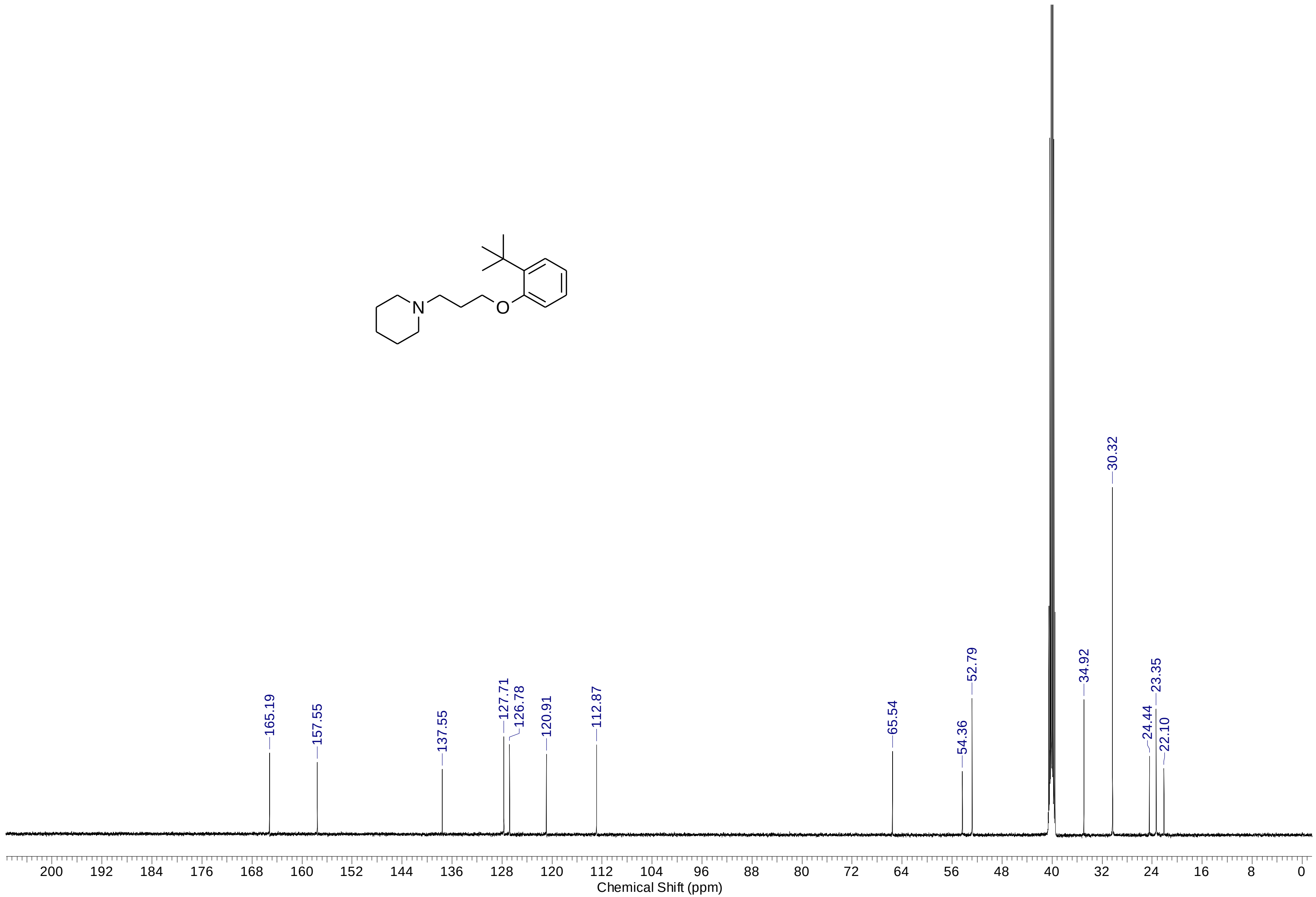

^{13}C NMR spectrum of 1-(3-(2-(*tert*-butyl)phenoxy)propyl)piperidine hydrogen oxalate (**24**)

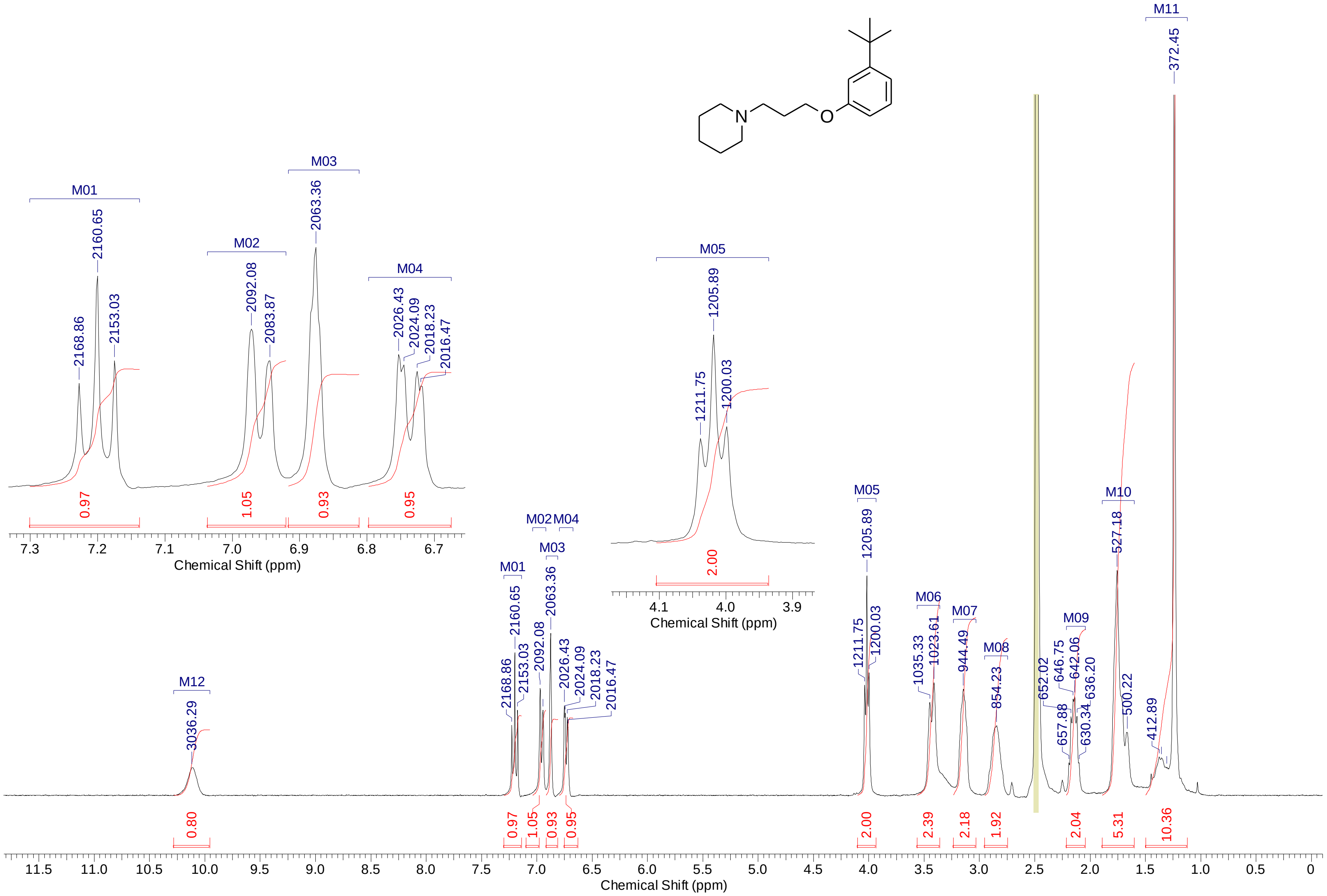

^{1}H NMR spectrum of 1-(3-(3-(*tert*-butyl)phenoxy)propyl)piperidine hydrogen chloride (**25**)

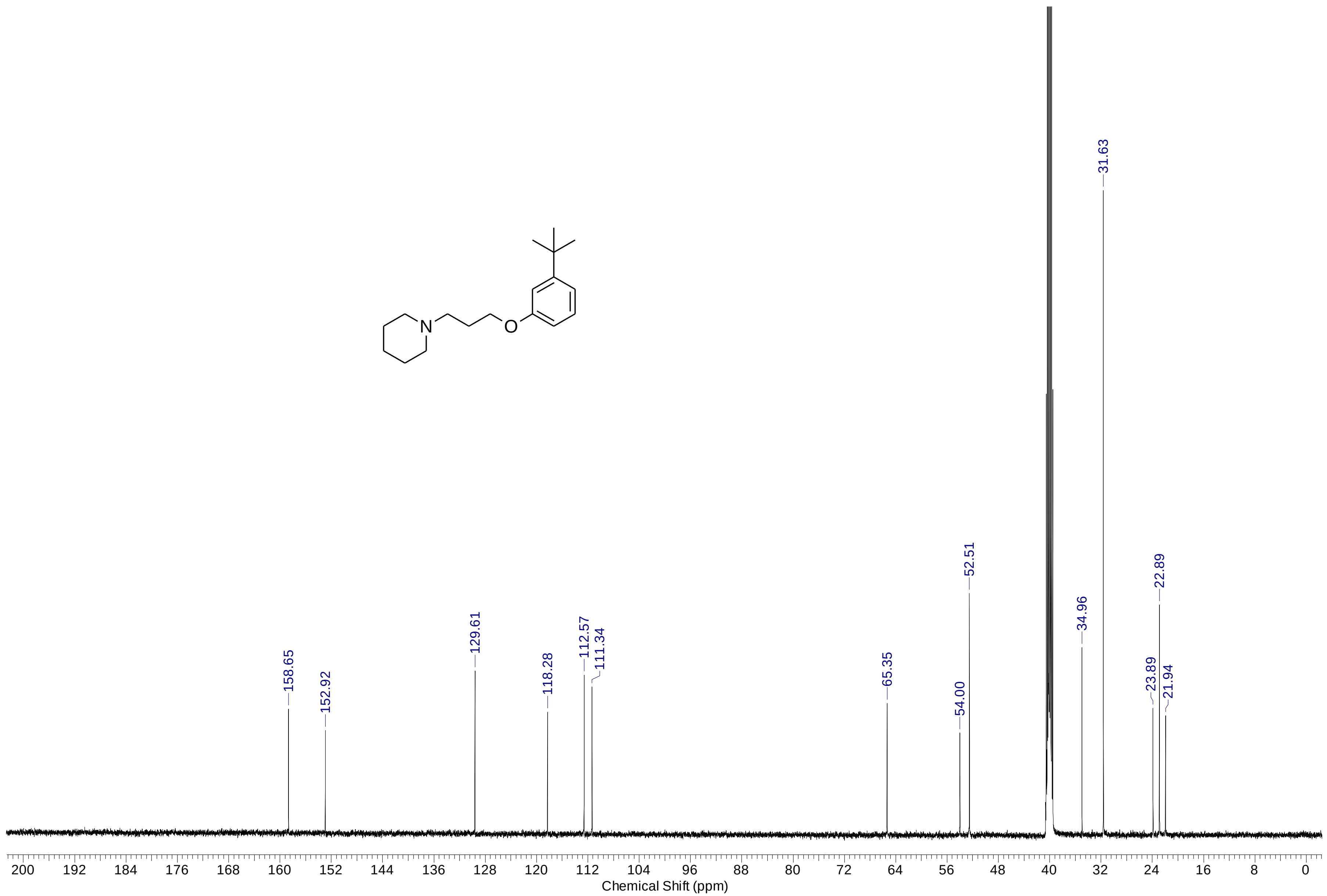

^{13}C NMR spectrum of 1-(3-(3-(*tert*-butyl)phenoxy)propyl)piperidine hydrogen chloride (**25**)

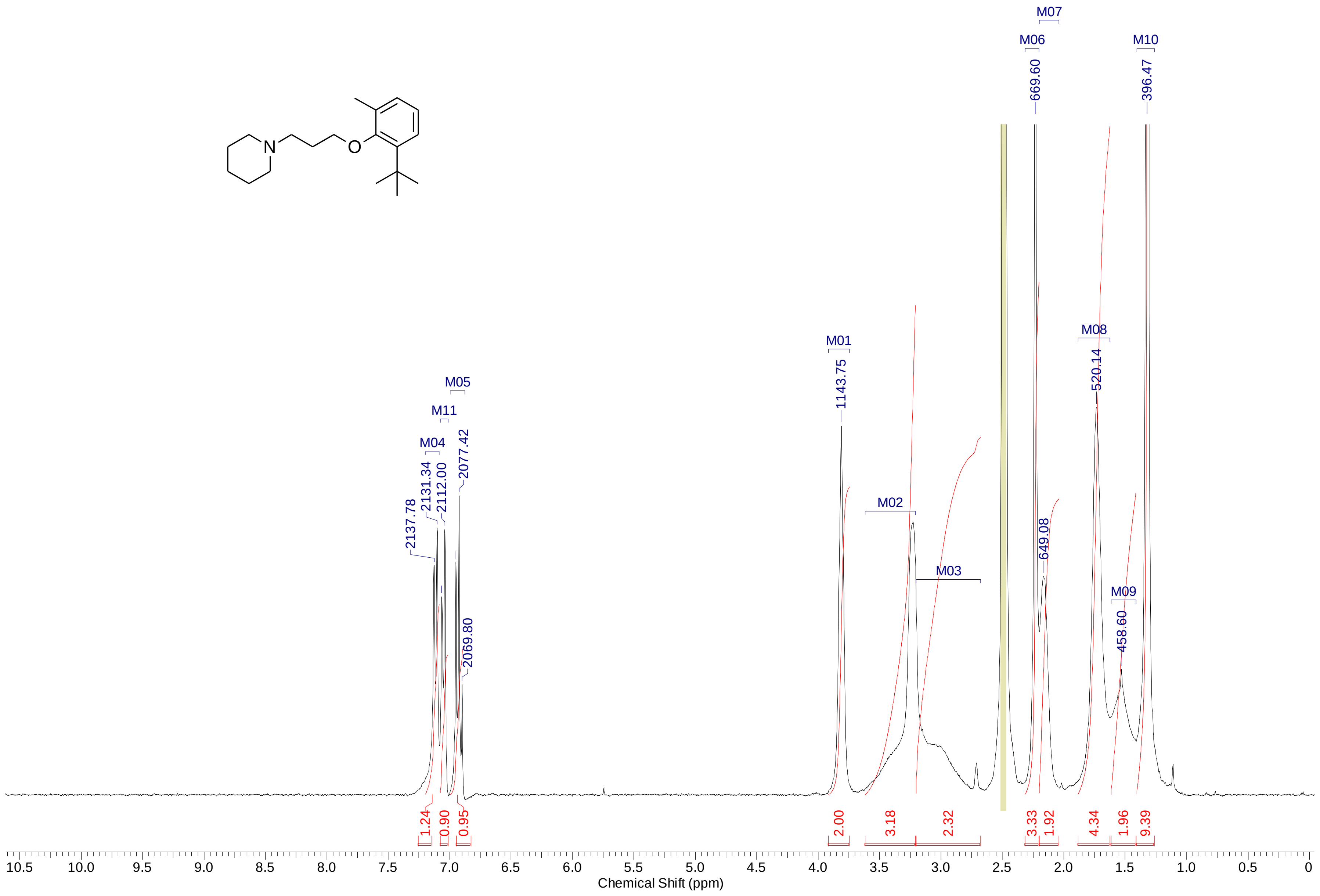

^{1}H NMR spectrum of 1-(3-(2-(*tert*-butyl)-6-methylphenoxy)propyl)piperidine hydrogen oxalate (**26**)

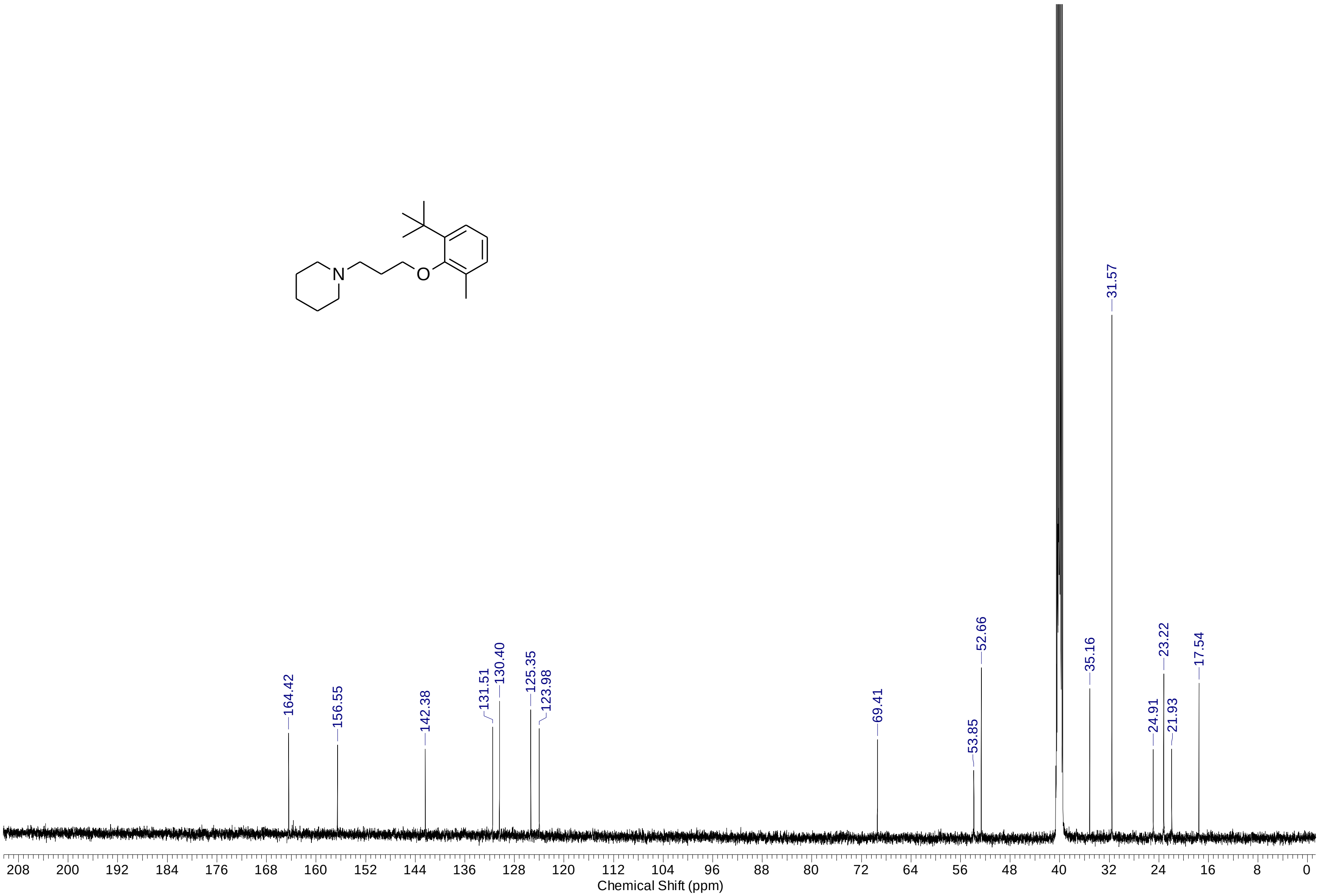

^{13}C NMR spectrum of 1-(3-(2-(*tert*-butyl)-6-methylphenoxy)propyl)piperidine hydrogen oxalate (**26**)

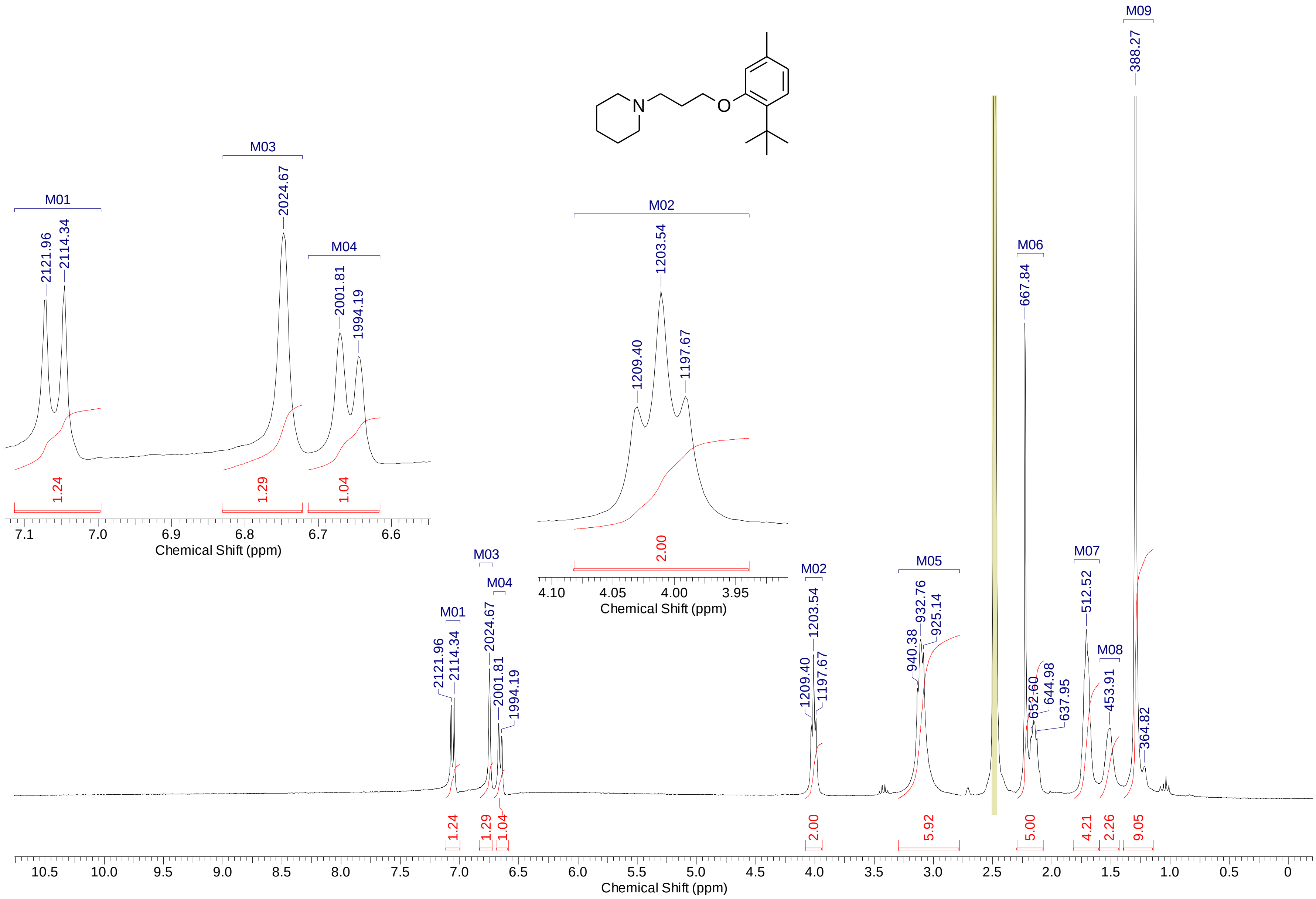

^{1}H NMR spectrum of 1-(3-(2-(*tert*-butyl)-5-methylphenoxy)propyl)piperidine hydrogen oxalate (**27**)

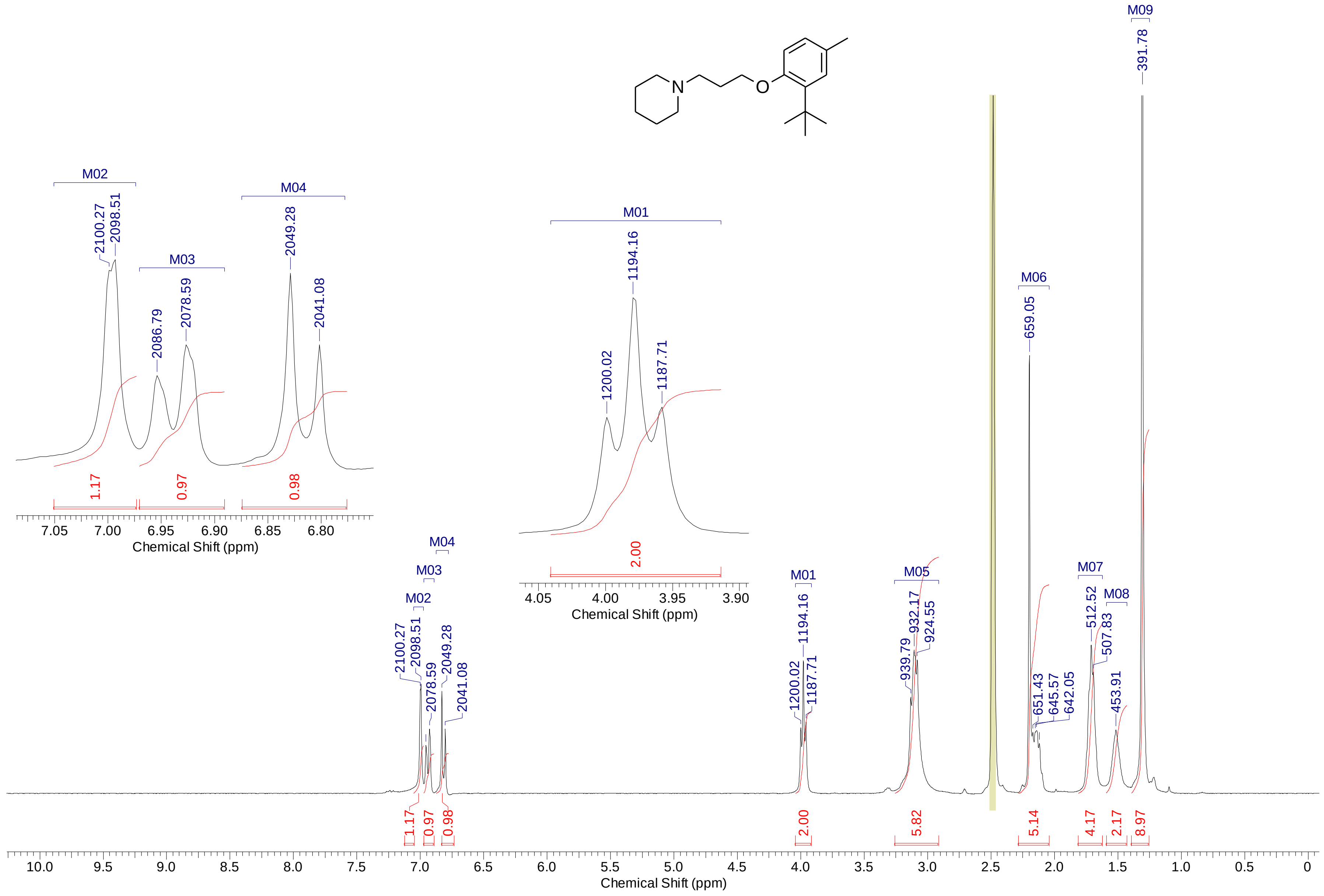

^{1}H NMR spectrum of 1-(3-(2-(*tert*-butyl)-4-methylphenoxy)propyl)piperidine hydrogen oxalate (**28**)

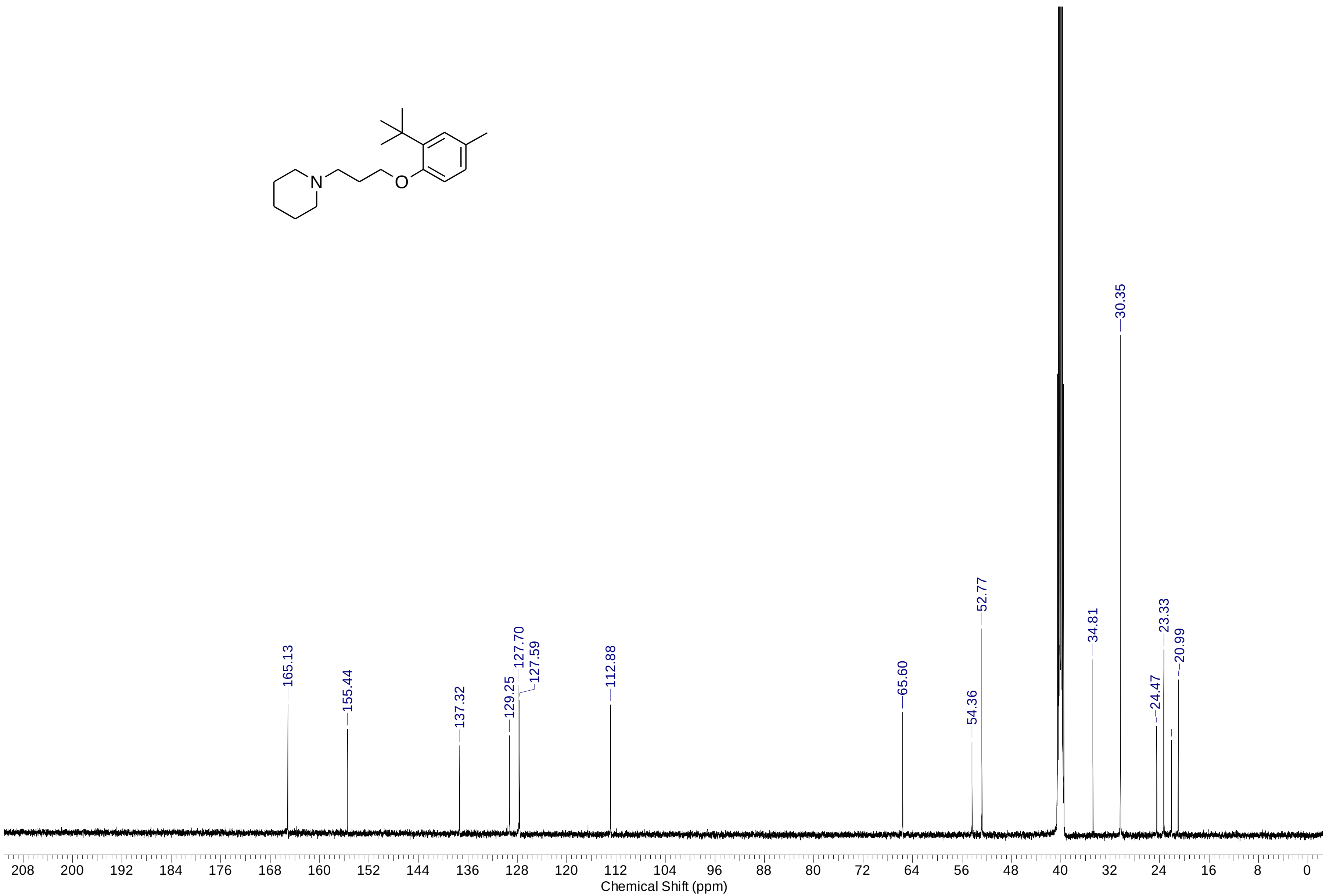

^{13}C NMR spectrum of 1-(3-(2-(*tert*-butyl)-4-methylphenoxy)propyl)piperidine hydrogen oxalate (**28**)

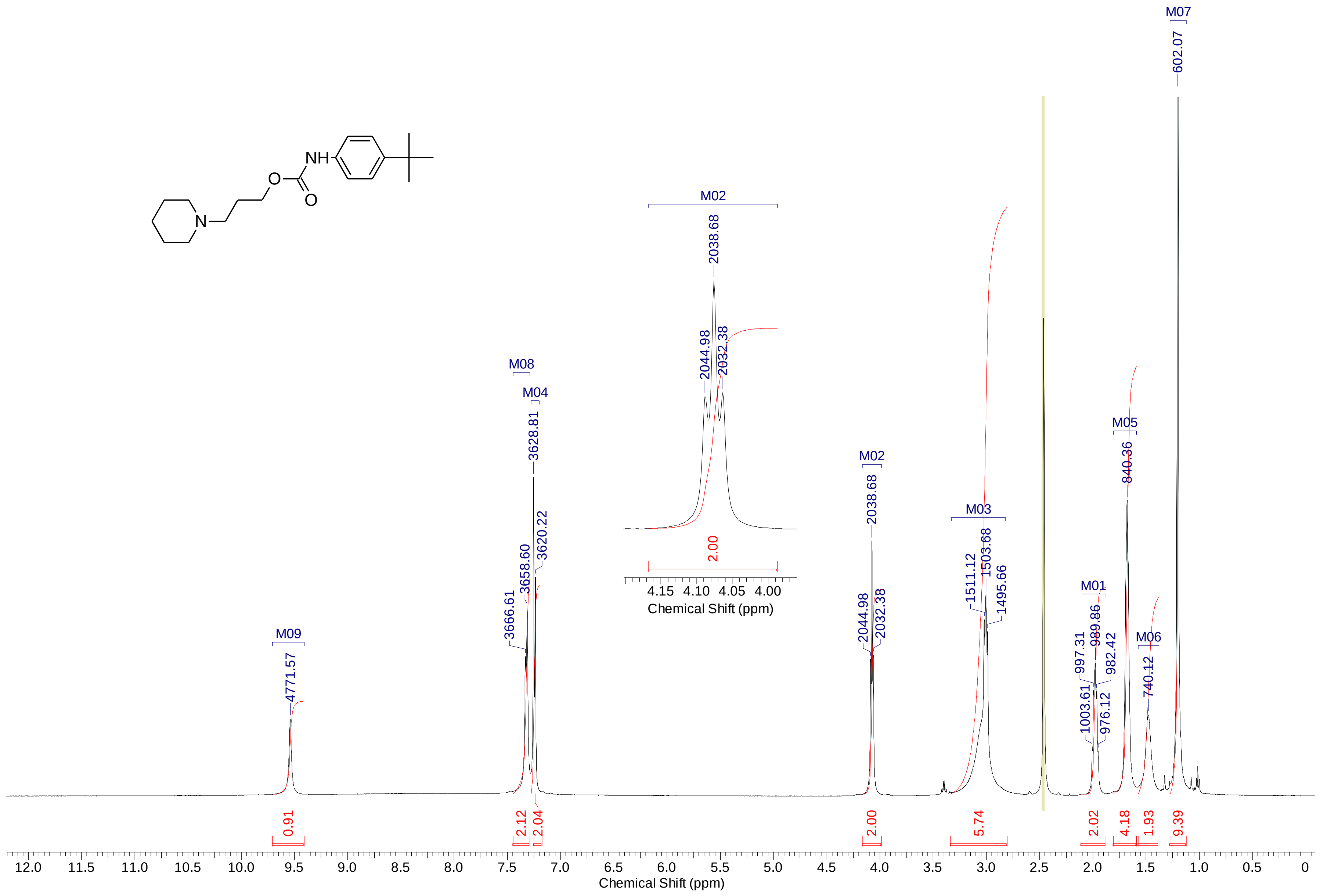

^{1}H NMR spectrum of 3-(piperidin-1-yl)propyl (4-(*tert*-butyl)phenyl)carbamate hydrogen oxalate (**29**

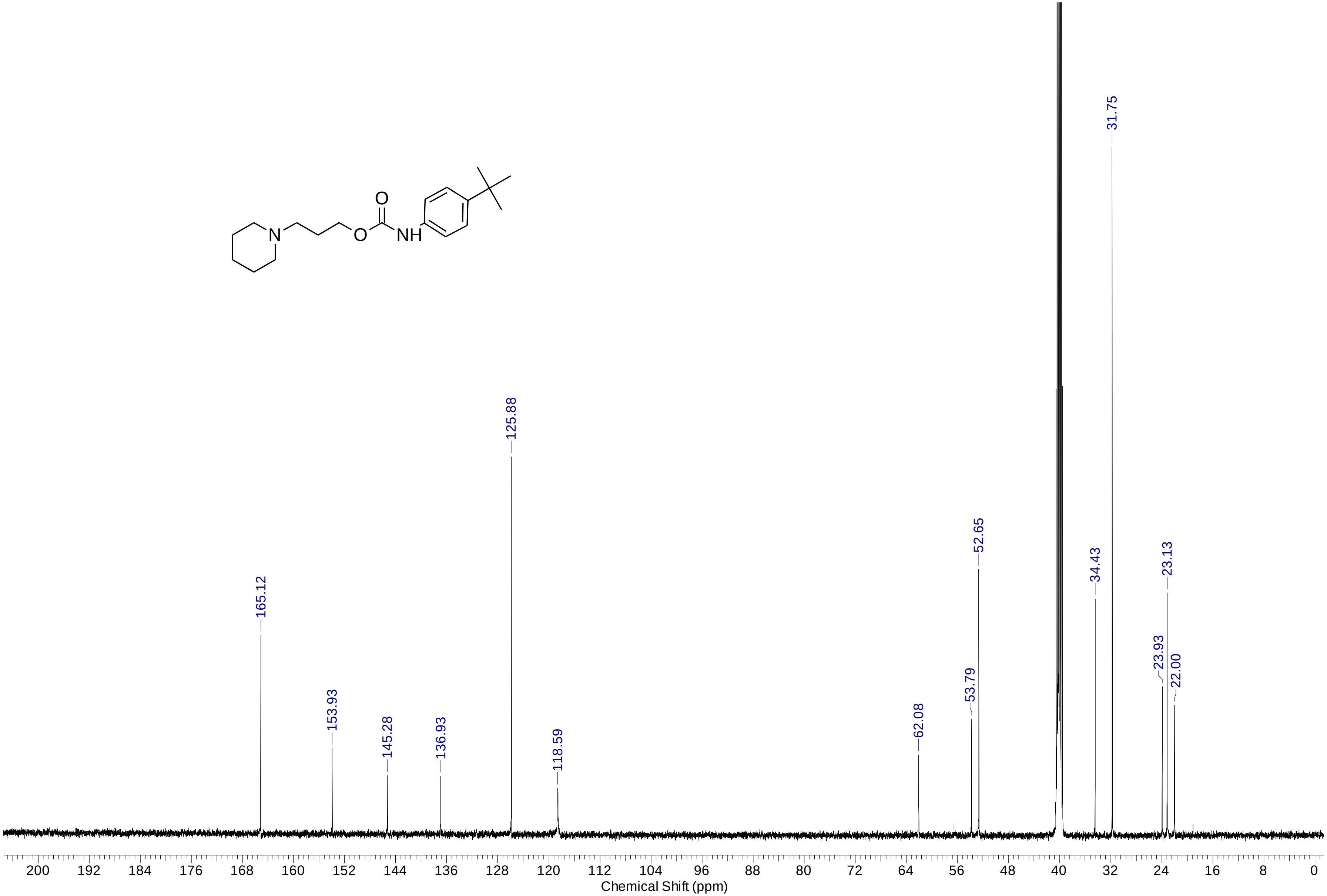

^{13}C NMR spectrum of 3-(piperidin-1-yl)propyl (4-(*tert*-butyl)phenyl)carbamate hydrogen oxalate (**29**)
